# Dissecting functional components of reproductive isolation among closely related sympatric species of the *Anopheles gambiae* complex

**DOI:** 10.1111/eva.12517

**Published:** 2017-10-05

**Authors:** Marco Pombi, Pierre Kengne, Geoffrey Gimonneau, Billy Tene‐Fossog, Diego Ayala, Colince Kamdem, Federica Santolamazza, Wamdaogo Moussa Guelbeogo, N'Falé Sagnon, Vincenzo Petrarca, Didier Fontenille, Nora J. Besansky, Christophe Antonio‐Nkondjio, Roch Kounbobr Dabiré, Alessandra della Torre, Frédéric Simard, Carlo Costantini

**Affiliations:** ^1^ Dipartimento di Sanità Pubblica e Malattie Infettive Università di Roma “Sapienza” Rome Italy; ^2^ Istituto Pasteur Italia‐Fondazione Cenci‐Bolognetti Rome Italy; ^3^ Institut de Recherche pour le Développement (IRD) UMR MIVEGEC (University of Montpellier, CNRS 5290 IRD 224) Centre IRD de Montpellier Montpellier France; ^4^ Organisation de Coordination pour la lutte contre les Endemies en Afrique Centrale (OCEAC) Yaoundé Cameroon; ^5^ CIRAD UMR INTERTRYP Montpellier France; ^6^ Centre International de Recherches Médicales de Franceville Franceville Gabon; ^7^ Department of Entomology University of California Riverside CA USA; ^8^ Centre National de Recherche et Formation sur le Paludisme (CNRFP) Ouagadougou Burkina Faso; ^9^ Institut Pasteur du Cambodge Phnom Penh Cambodia; ^10^ Eck Institute for Global Health & Department of Biological Sciences University of Notre Dame Notre Dame IN USA; ^11^ Institut de Recherche en Sciences de la Santé (IRSS) Bobo‐Dioulasso Burkina Faso

**Keywords:** ecological speciation, hybridization, postmating, premating, reproductive isolation, species complex

## Abstract

Explaining how and why reproductive isolation evolves and determining which forms of reproductive isolation have the largest impact on the process of population divergence are major goals in the study of speciation. By studying recent adaptive radiations in incompletely isolated taxa, it is possible to identify barriers involved at early divergence before other confounding barriers emerge after speciation is complete. Sibling species of the *Anopheles gambiae* complex offer opportunities to provide insights into speciation mechanisms. Here, we studied patterns of reproductive isolation among three taxa, *Anopheles coluzzii*,* An. gambiae s.s*. and *Anopheles arabiensis*, to compare its strength at different spatial scales, to dissect the relative contribution of pre‐ versus postmating isolation, and to infer the involvement of ecological divergence on hybridization. Because F1 hybrids are viable, fertile and not uncommon, understanding the dynamics of hybridization in this trio of major malaria vectors has important implications for how adaptations arise and spread across the group, and in planning studies of the safety and efficacy of gene drive as a means of malaria control. We first performed a systematic review and meta‐analysis of published surveys reporting on hybrid prevalence, showing strong reproductive isolation at a continental scale despite geographically restricted exceptions. Second, we exploited our own extensive field data sets collected at a regional scale in two contrasting environmental settings, to assess: (i) levels of premating isolation; (ii) spatio/temporal and frequency‐dependent dynamics of hybridization, (iii) relationship between reproductive isolation and ecological divergence and (iv) hybrid viability penalty. Results are in accordance with ecological speciation theory predicting a positive association between the strength of reproductive isolation and degree of ecological divergence, and indicate that postmating isolation does contribute to reproductive isolation among these species. Specifically, only postmating isolation was positively associated with ecological divergence, whereas premating isolation was correlated with phylogenetic distance.

## 1 INTRODUCTION

Speciation proceeds by the progressive establishment of reproductive isolation among genetically distinct populations. Reproductive isolation results from the combined effect of all barriers to gene flow, which can be conveniently classified into three functional components (Mallet, 2006): (i) natural selection against immigrants from alternative ecological niches; (ii) sexual isolation due to assortative mating or fertilization; and (iii) selection against zygotes formed when hybridization does occur. The latter mechanism can be further partitioned according to whether selection results from the presence of intrinsic genetic incompatibilities, expressed in the form of hybrid sterility or inviability independent of the environment, or rather from inferior hybrid genotypes as a result of ecological mechanisms, a process also known as extrinsic postmating isolation. Barriers to gene flow are therefore multifarious, with the accumulation of multiple mechanisms in the course of population divergence gradually producing an increase in the nature and strength of isolation. Accordingly, time since lineage divergence is a reasonable predictor of the strength of reproductive isolation, as closely related taxa exhibit weaker isolation compared to distantly related ones (Coyne & Orr, 1998; Orr & Coyne, 1989). While this relationship holds true noisily, several evolutionary processes can distort it when considering closely related taxa issuing from recent adaptive radiations: at this phylogenetic level, the strength of reproductive isolation can be modulated by geographical, historical or ecological factors that weaken or strengthen the barriers against gene flow.

Explaining how and why reproductive isolation evolves and, in particular, determining which forms of reproductive isolation have the largest impact on the process of divergence, are major goals in the study of speciation. The relative importance of different categories of reproductive isolation in the process of speciation, however, has been the focus of much debate (Coyne & Orr, 1989, 1997; Rice & Hostert, 1993; Sobel, Chen, Watt, & Schemske, 2010). One of the main issues lies in the sequential nature of isolation mechanisms at different temporal scales. From a chronological perspective, measures of the strength of reproductive isolation should account for the fact that postmating barriers intervene after premating barriers have already filtered the potential for hybridization (Sobel & Chen, 2014). Moreover, several lines of evidence show that the strength of postmating isolation varies in relation to the degree of ecological divergence, whereas premating isolation follows the general pattern of increase with time since lineage divergence (Turelli, Lipkowitz, & Brandvain, 2014). At an evolutionary level, reproductive barriers continue to accumulate even after speciation is complete, so that the strength and nature of isolation in extant species may be radically different from that existing during early divergence (Sobel & Streisfeld, 2015). The study of recent adaptive radiations of partially isolated taxa allows identifying barriers involved in their initial divergence. This permits the separation of these barriers from other factors emerging when speciation is complete. Hence, studies of recently diverged taxa provide the most accurate picture of the barriers involved in speciation, and comparative analysis of multiple, recently diverged species may be the only way to assess the order in which barriers typically arise (Sobel et al., 2010). In this respect, isomorphic members of the afro‐tropical *Anopheles gambiae sensu lato* (*s.l*.) complex, which includes eight members exhibiting different degrees of reproductive isolation and ecological divergence, offer an opportunity to provide insights into the mechanisms of emergence of reproductive isolation and hybridization, whose implications are not only of interest to evolutionary biologists but also of importance for malaria control. Current malaria control strategies are based on insecticides, which exert strong selective pressures on vector populations, affecting their ecology and behaviour (Kitau et al., 2012; Russell et al., 2011). Innovative vector‐borne disease control strategies are based on population suppression or replacement by genetic technologies, which rely on driving genes of interest into natural mosquito populations. In both instances, adaptive introgression through hybridization can accelerate the evolution of insecticide resistance or gene drive across semipermeable species barriers (Norris et al., 2015).

Three species of the complex—*An. gambiae sensu stricto* (*s.s*.), *Anopheles coluzzii* and *Anopheles arabiensis*—have the widest range of distribution and represent the most efficient vectors of human malaria in sub‐Saharan Africa, where they are responsible for hundreds of millions of infections and hundreds of thousands of deaths (WHO, 2016). The sibling species *An. gambiae s.s*. and *An. coluzzii* are believed to have split about 540,000 years ago and represent the most recent radiation in the species complex (Fontaine et al., 2015; Kamali, Xia, Tu, & Sharakhov, 2012). They correspond to two assorting taxonomic units provisionally named “molecular forms S and M” defined based on fixed mutations on the chromosome‐X‐linked rDNA intergenic spacer (IGS) (della Torre et al., 2001) and have only recently received formal Linnaean nomenclature (Coetzee, Wilkerson, della Torre, Coulibaly, & Besansky, 2013), based on evidence showing that their genomes contain regions of differentiation resulting in exclusive taxonomic clustering across much of their shared geographical range (Lawniczak et al., 2010; Neafsey et al., 2010; Reidenbach et al., 2012). To avoid confusion in the nomenclature, we chose to use the definition “*An. gambiae* (Giles)” for both taxa collectively, and “*An. gambiae s.s*.” with reference to the single taxon formerly named as molecular form S. Genomic studies have clearly demonstrated that *An. gambiae s.s*. and *An. coluzzii* ancestor separated from other species in the group approximately 2 million years ago (Ma) and that the two species are distantly related to *An. arabiensis* (Fontaine et al., 2015; Kamali et al., 2012). The latter species can be distinguished from *An. gambiae* (Giles) by diagnostic fixed paracentric chromosomal inversions on the X heterosome (Coluzzi & Sabatini, 1967; White, 1971), as well as by diagnostic DNA‐based differences in the IGS region (Scott, Brogdon, & Collins, 1993). Despite a history of extensive introgression among the three species (Fontaine et al., 2015), these are behaviourally, physiologically, ecologically and epidemiologically distinct and segregate along major ecoclimatic gradients at a continental scale. Overall, *An. arabiensis* and *An. gambiae s.s*. are sympatric across sub‐Saharan Africa, but the former species dominates in more xeric areas and at higher altitudes, while it is virtually absent in the moist Guineo‐Congolese rainforest block of Western and Central Africa (Coetzee, Craig, & Le Sueur, 2000; White, 1974). *Anopheles coluzzii* is only found west of the Rift Valley where it extensively overlaps with *An. arabiensis* in savannah areas (Costantini et al., 2009). Conversely, there is evidence of strong segregation between *An. gambiae s.s*. and *An. coluzzii*, with a prevalence of the latter in ecologically more complex and more stable anthropogenic larval habitats (Gimonneau et al., 2012; Kamdem et al., 2012). Overall, therefore, *An. coluzzii* appears ecologically more similar to *An. arabiensis* compared to *An. gambiae s.s*. (Costantini et al., 2009), suggesting that *An. coluzzii* is likely to have diverged from *An. gambiae s.s*. and to have invaded, during this process, the ecological niche of *An. arabiensis*.

All the siblings of the complex freely interbreed in laboratory cages; however, they show different degrees of postzygotic incompatibilities. The hybrid progeny resulting from crossing *An. arabiensis* with *An. gambiae* (Giles) (i.e., not distinguishing between *An. gambiae s.s*. and *An. coluzzii*) are generally fit and viable, but hybrid males are sterile, and backcross females show reduced fertility due to several incompatible alleles (Slotman, della Torre, Calzetta, & Powell, 2005; Slotman, della Torre, & Powell, 2004). The rarity of *An. arabiensis × gambiae* (Giles) individuals in natural field populations (Touré et al., 1998; White, 1971) is probably a consequence of strong (albeit incomplete) premating isolation and of selection acting against these hybrids (Mallet, 2005). Although experimental evidence is lacking, premating behavioural differences among *An. arabiensis*,* An. coluzzii* and *An. gambiae s.s*. imperfectly enforce isolation, given that mixed swarms are sometimes found in nature (Dabire et al., 2013). Conversely, there is no evidence of genetic incompatibilities in experimental crosses between *An. coluzzii* with *An. gambiae s.s*.: hybrids are viable and fertile, with no obvious loss in fitness in a laboratory setting (Diabaté et al., 2005, 2009; Hahn, White, Muir, & Besansky, 2012; Sawadogo et al., 2014). Assortative mating between *An. coluzzii* and *An. gambiae s.s*. is imperfectly maintained in nature (Tripet et al., 2001) and periodically breaks down, resulting in extensive hybridization (Caputo et al., 2011; Costantini et al., 2009; Lee et al., 2013; Oliveira et al., 2008) and in detectable levels of introgression and current gene flow (Marsden et al., 2011; Reidenbach et al., 2012; Weetman, Wilding, Steen, Pinto, & Donnelly, 2012). Several premating behavioural mechanisms such as spatial segregation of mating swarms (Diabaté et al., 2009), complex short‐range acoustic recognition responses (Pennetier, Warren, Dabiré, Russell, & Gibson, 2010; Simões, Gibson, & Russell, 2017) and lags in circadian activity associated with reproductive behaviour (Sawadogo et al., 2013) might contribute to diminish heterospecific inseminations. It is plausible that reduced hybrid fitness mediated by extrinsic ecological factors, as opposed to intrinsic genetic incompatibilities, may further contribute to reproductive isolation, making these species another example of ecological speciation.

In recent species radiations like this one, ongoing hybridization and introgression may be a means of spreading adaptive traits across species boundaries (Fontaine et al., 2015). In the case of the *An. gambiae* species complex of malaria vectors, this may entail the exchange of genes that have an important bearing on aspects of vectorial capacity, including blocks of genes inside chromosomal inversions that have been implicated in aridity tolerance, and insecticide or parasite resistance genes (Clarkson et al., 2014; Djogbénou et al., 2008; Mancini et al., 2015; Norris et al., 2015; Sharakhov et al., 2006; White et al., 2011). The existence of interspecific gene flow even between nonsister species potentially accelerates adaptive change and speeds the development of resistance to vector control tools, but also may beneficially broaden the target populations susceptible to gene drive strategies aimed at reducing malaria transmission (Knols, Bossin, Mukabana, & Robinson, 2007; Scott, Takken, Knols, & Boete, 2002). For this reason, an understanding of the dynamics and the ecological conditions favouring hybridization between taxa in this group is of paramount importance.

Here, we take advantage of published data at continental scale as well as of extensive original field data sets collected at a regional scale in two contrasting environmental settings to dissect the different functional components of reproductive isolation among *An. coluzzii*,* An. gambiae s.s*. and *An. arabiensis* (from pre‐ to postmating isolation and ecological divergence) at different spatial scales. Altogether, our results point to the dynamic nature of reproductive isolation and show how different genetic, behavioural and ecological components interact in a complex way to modulate its strength.

## 2 MATERIALS AND METHODS

### 2.1 Frequency of hybrids in field populations at a continental scale: a meta‐analysis

We performed a systematic search and review of field studies indexed in the publication repository PubMed (http://www.ncbi.nlm.nih.gov/pubmed). We looked for studies reporting on the occurrence and frequency of *An. gambiae s.s*. and *An. arabiensis* populations in sympatry that were published between 1964 and March 2013. Articles were searched using the keywords “gambiae AND larvae,” or “gambiae AND adults,” in association with any of the following keywords: “distribution,” “frequency,” “identification,” “sympatry,” “hybrid,” or “molecular form.” From the results of the search, we retrieved the following information: country of collection, total number of specimens collected (females), number of hybrids identified, number of localities where the specimens were collected, and the name and geographical coordinates of the sites, whenever available. Articles reporting about specimens collected as larvae that were subsequently grown in insectaries were not included in the analysis to avoid biases due to rearing in artificial conditions.

As the status and nomenclature of the taxonomic subdivisions within *An. gambiae s.s*. have changed, we chose to classify results from articles that did not distinguish between the M and S molecular forms of *An. gambiae s.s*. (e.g., all those published before 2001) as *An. gambiae* (Giles), and results from studies referring to the M and S forms as *An. coluzzii* and *An. gambiae s.s*., respectively. When only the chromosomal form—not the molecular form—was defined, results were classified as *An. gambiae* (Giles) as the taxonomic subdivisions within each of these two classification systems do not coincide.

Four data sets were assembled, two sets to study the prevalence of *An. arabiensis *× *gambiae* (Giles) hybrids in larval and adult samples, and two sets for *An. coluzzii *× *gambiae s.s*. larval and adult hybrids, respectively. A meta‐analysis was performed on each data set to test whether the prevalence of hybrids was homogeneous across studies, that is whether there was a “study effect” that might be related to geographical heterogeneities. In cases when there was no study effect, studies could be combined to provide an overall estimate of the prevalence of hybrids. Homogeneity across studies was assessed by the Cochran *Q* and *I* ^2^ statistics (R Core Team, 2013; Viechtbauer, 2010). Whenever the *p*‐value of Cochran *Q* was greater than 5% and it was associated with a value of *I* ^2^ ≤ 25%, the studies were considered homogeneous and the relative frequency of hybrids was calculated considering a fixed‐effects combined value. Conversely, if the studies were significantly heterogeneous, a random‐effects combined value was calculated. Under the fixed‐effects model, it is assumed that the true effect is the same in all studies, while under the random‐effects model allowance is made for the true effect to vary across studies (Higgins, Thompson, Deeks, & Altman, 2003).

### 2.2 Assessment of premating isolation

In anopheline mosquitoes, after copulation male sperms are stored by the female inside a spermatheca, from which they are later retrieved to fertilize eggs during oviposition (Clements, 1999). Thus, as members of the *An. gambiae* complex are essentially monandrous or nearly so (Tripet et al., 2001), it is possible to know whether an individual female copulated with a conspecific or heterospecific male by molecularly typing the sperm isolated from the spermatheca. This allows investigating the strength of sexual isolation among members of the complex across different populations. Accordingly, we assessed the degree of pairwise assortative mating by counting the frequency of homospecific and heterospecific copulation in three populations of *An. arabiensis*,* An. coluzzii* and *An. gambiae s.s*. from central Burkina Faso and a population from southern Cameroon: indoor‐resting female mosquitoes were collected by pyrethroid spray‐sheet catches from the neighbouring villages of Kougoulapaka (12.13N, 1.96W), Koukoulou (12.09N, 1.85W) and Salbisgo (12.19N, 2.01W) at the end of the 2006 rainy season (30 September–21 October) in central Burkina Faso, and in Nkolbisson (site 9 in fig. 1E of Kamdem et al., 2012) and neighbouring Leboudi (3°54′14″N, 11°26′56″E) between November 2008 and February 2009 in the capital of Cameroon, Yaoundé.

The choice of locations was justified by several reasons: first, the sympatric occurrence of the taxa in comparable frequencies within each population, as expected from species distribution models developed previously for both countries (Costantini et al., 2009; Kamdem et al., 2012; Simard et al., 2009). This condition maximizes the opportunity for contact between taxa (and indeed was found to correlate with the highest frequency of hybrids in the population; cf. Section 3). Second, the high prevalence of *An. coluzzii* × *gambiae s.s*. hybrids recorded from small samples collected in two of these localities (Koukoulou and Kougoulapaka) during a countrywide survey carried out the year before sampling (Costantini et al., 2009). Third, the different geographical mode of contact between *An. coluzzii* and *An. gambiae s.s*. in the two countries: in the arid savannah of Burkina Faso, the two taxa are essentially sympatric (Costantini et al., 2009), whereas in the forested area of Yaoundé in southern Cameroon *An. coluzzii* and *An. gambiae s.s*. are parapatric as they segregate steeply along urbanization clines (Kamdem et al., 2012). Finally, the two environmental settings reflect different genetic backgrounds of the two species; in particular, chromosomal polymorphisms are markedly different in these two ecogeographical areas. While we could not explicitly test for the individual effect on sexual isolation of each of these variables (degree of overlap, habitat or chromosomal polymorphism) due to collinearity and lack of replication, we included a differentiated panel of populations to study general mechanisms of premating isolation.

Following the protocol of Tripet et al. (2001), adult females morphologically identified as *An. gambiae s.l*., or their spermatheca isolated from the rest of the carcass, were individually preserved in 1.5‐ml microtubes containing 70% ethanol for ≥2 days to agglutinate the sperm in the form of a bundle. Mosquito carcasses were put in individual microtubes containing a desiccant (silica gel). After careful separation of the sperm mass from the chitinous shell of the spermatheca under a dissecting microscope, the sperm bundle was transferred in a microtube for molecular analysis. The identity of the female and associated paternity of the sperm were obtained by molecular identification following a PCR‐RFLP protocol (Fanello et al., 2003) performed on a leg from each mosquito carcass and from DNA extracted from the sperm bundle with 5% Chelex (Walsh, Metzger, & Higuchi, 1991) or the Qiagen “DNeasy” extraction kit according to manufacturer's instructions. Specimens that did not return interpretable bands by the direct PCR were further processed by extraction of DNA from two legs by CTAB (Cornel & Collins, 1996), and the PCR repeated.

We have assessed the degree of assortative mating from indices of pair sexual isolation (*I*_PSI_) using the software JMATING (Carvajal‐Rodriguez & Rolan‐Alvarez, 2006). *I*_PSI_ is a safe estimator of sexual isolation for field samples, as it is not affected by small variances across different scenarios of frequencies of the taxa considered, nor by mate propensity, that is the intrinsic tendency for some individuals/populations/taxa to mate more frequently than others (Pérez‐Figueroa, Cruz, Carvajal‐Rodríguez, Rolán‐Alvarez, & Caballero, 2005). Mean bootstrap values, standard deviations and two‐tail probabilities for rejecting the null hypothesis (*I*_PSI_ = 0, i.e., random mating) were calculated by bootstrap (100,000 resamplings, also for premating frequencies).

If two taxa mate partially at random, the frequency of homogamous mating depends on the probability of meeting between sexes of the same taxon. This probability depends on the frequency of individuals of the focal taxon in the whole population. The higher the frequency, the higher is the probability that conspecific individuals of the focal taxon will meet, and consequently levels of heterogamy will be lower. To test this hypothesis, we have fitted generalized linear models to the *I*_PSI_ indices using the relative frequency in the population of the pair of taxa whose sexual isolation was under scrutiny as a covariate; the focal taxa were fitted as a factor and its interaction with the covariate tested in an ANCOVA‐like arrangement *I*_PSI_ = FREQUENCY * TAXA, which is formally:

$$I_{\mathrm{PSI}}^{ijk}=\alpha_{ij}+\beta_{ij}p_{ijk}+\epsilon$$

where the pair sexual isolation index of species pair *i*,* j* at location *k* is equal to the sum of linear components, with α and β representing the intercept and slope of the linear relationship, *p*_*ijk*_ the frequency at location *k* of species *i* relative to species *j*, and ε are sampling errors around the regression line. We have assumed that errors are distributed normally and fitted GLMs with identity link functions. We tested the statistical significance of the interaction and main effects of the FREQUENCY and TAXA variables by likelihood‐ratio tests on the change in deviance associated with removal of nested model terms, and searched for the minimal adequate model by means of the Akaike information criterion. While a parabolic relationship between *I*_PSI_ and frequency, with a minimum at intermediate values and two local maxima at the opposite ends of the covariate scale, may better represent the dependence of sexual isolation on the relative abundance of the focal taxa, the limited size of our data set and range of observed *p*_*ijk*_ values oriented our analysis towards a linear model, which can approximate parabolic‐like relationships at one end of the covariate scale.

### 2.3 Spatial and temporal patterns of hybridization in a “contact zone”

We exploited the large data set available along an urbanization gradient in the capital of Cameroon—Yaoundé (Kamdem et al., 2012), to analyse in more detail the spatial and temporal distribution of *An. coluzzii* × *gambiae s.s*. larval and adult hybrids. The aim was to investigate whether hybridization clustered in space and/or time at a microgeographical scale, according to the relative frequency and population dynamics of the parental species. This area falls out of the geographical distribution range of *An. arabiensis*, so hybrids resulting from hybridization with this species could not be observed.

A systematic search for *An. gambiae* (Giles) indoor‐resting adults and larvae in nearby breeding sites was carried out during one week monthly, from May 2008 to April 2009 along a 18‐km transect running from the central area of Yaoundé to the nearest westerly rural villages. Sampling locations were separated by ~1 km each. Adult mosquitoes were collected by spraying an insecticide aerosol inside the sleeping rooms of 1,481 households, whereas larvae were collected by dipping from 3,421 aquatic habitats. Temporal clustering of hybridization was tested with the rank version of the von Neumann ratio test for randomness (Bartels, 1982) using the R package *lawstat* (Hui, Gel, & Gastwirth, 2008).

### 2.4 Frequency of hybrids at a regional scale in populations from Burkina Faso and Cameroon

Similarly, we aimed to investigate how the spatial distribution of hybridization relates to the relative frequency of the parental species at a larger (regional) spatial scale using extensive data sets collected in Burkina Faso and Cameroon during independent surveys performed between 2005 and 2012 in about 600 localities sampled within the framework of several research projects. Mosquitoes were collected by any of two methods (indoor‐resting adult females caught by insecticide spray‐sheet collections, and larvae by dipping), identified morphologically as *An. gambiae s.l*. and preserved in microtubes containing either a desiccant (silica gel) or 70% ethanol. Molecular identification of individual specimens was performed using one of several possible diagnostic PCR protocols (Fanello et al., 2003; Favia, Lanfrancotti, Spanos, Sidén‐Kiamos, & Louis, 2001; Santolamazza et al., 2008). It is worth noting that all the samples from Burkina Faso used in this analysis are from indoor‐resting mosquitoes, a feature that relieves difficulties of interpretation due to the potential presence of a cryptic subgroup of *An. gambiae* (Giles) in this study area (Riehle et al., 2011) (for more details, see Appendix S1).

To verify whether the prevalence of hybrids changed as a function of the relative abundance of the parental taxa in the population, we have fitted generalized additive models (GAM) assuming that the frequency of hybrids ${\left\langle A\times B\right\rangle }_{i}$ in sample *i* containing ${\left\langle A+B+A\times B\right\rangle }_{i}$ individuals follows a binomial distribution with hybridization rate *h*_*i*_. This is ${\left\langle A\times B\right\rangle }_{i}∼\text{Binomial}\left({\left\langle A+B+A\times B\right\rangle }_{i},h_{i}\right)$, where *A* and *B* represent the absolute frequencies of the parental species. The models assume that the logarithm of the odds of the probability of hybridization *h*_*i*_ is a linear function of the relative frequency of the parental taxa *p*_*i*_ in each locality, that is: ${\text{log}}_{e}\left(h_{i}/\left(1-h_{i}\right)\right)=\alpha +\phi \left(p_{i}\right)+\varepsilon$, where ϕ is a nonparametric smoothed function, and ε represent the sampling error. We estimated *h*_*i*_ and *p*_*i*_ from the sample estimates $\hat{h_{i}}={\left\langle A\times B\right\rangle }_{i}/{\left\langle A+B+A\times B\right\rangle }_{i}$ and $\hat{p_{i}}={\left\langle A\right\rangle }_{i}/{\left\langle A+B\right\rangle }_{i}$.

Because hybridization in these regions is rare, we did not take into account the frequency of hybrids in the denominator of $\hat{p_{i}}$. We fitted GAM with the R package *mgcv* (Wood, 2006), specifying a binomial error structure and a logit link function. This approach has the added benefit of weighing each locality by its sample size. The statistical significance of the inclusion of the nonparametric smoothed function in the model was tested by the difference in AIC and likelihood‐ratio test associated with removal of the nonparametric term from the model.

### 2.5 Assessment of postmating isolation

**Figure 1:**
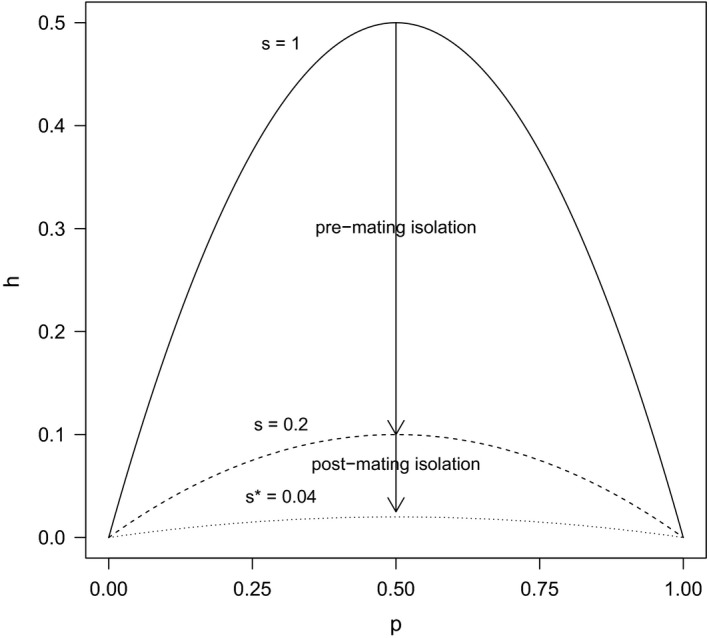
Contribution of premating versus postmating barriers towards the prevalence of hybrids (*h*) under a mode of frequency‐dependent hybridization; *p* is the proportion in the population of one of the two hybridizing species. The parameter *s* represents the proportional decrease in hybrids from Hardy–Weinberg expectations due to assortative mating—a measure of the strength of the pre‐mating barrier. When *s *= 1, mating is random; that is, there is no premating isolation. The parameter s* represents the proportional decrease in hybrids from Hardy–Weinberg expectations due to total reproductive isolation. The fraction s*/*s* denotes the proportion of viable hybrid zygotes, which accounts for the proportional decrease in hybrids from Hardy–Weinberg expectations due to postmating isolation. The example shows the disproportionate contribution of the premating barrier to total reproductive isolation when both *s* and s*/*s* are equal to 0.2, which is due to the sequential nature and different scaling properties of the two processes. Other cases, not shown, are equally possible

To assess the relative contributions of pre‐ versus postmating isolation, we modelled the prevalence of hybrids in a population in relation to the proportion of individuals that mate at random according to Hardy–Weinberg expectations: if all individuals of the two species in a population mate at random, the expected frequency *h* of hybrids, when these are fully viable, can be expressed by *h* = 2*pq*, with *p* representing the frequency of one of the parental taxa and *q* = 1 – *p*. This value represents the maximum expected prevalence of hybrids at any given frequency of the parental taxa (Figure 1).

If only a fraction *m* (≤1) of individuals in the population mate at random while the remaining proportion (1 – *m*) mate assortatively, the expected frequency of hybrids is reduced to *h* = 2*m* ^2^ *pq*. For comparative purposes, we express the coefficient *m* ^2^, representing the proportional decrease in hybrids from H–W expectations due to assortative mating, as *s* = *m* ^2^, so that the expected frequency of hybrids becomes *h* = 2*spq*; lower values of *s* correspond to stronger premating isolation. If only a fraction s*/*s* of those hybrids survive, their prevalence is further reduced to *h* = 2s*pq (Figure 1). Lower values of the fraction s*/*s* denote stronger postmating isolation relative to premating isolation; when s*/*s* = 1, s* = *s*, meaning that reproductive isolation is fully accounted for by the premating barrier (i.e., no postmating isolation). This model (hereafter the H–W model as a shorthand) can be useful to assess the degree of postmating isolation relative to premating isolation, expressed by the fraction s*/*s*, when independent estimates of these parameters can be obtained. The parameters *s* and s* of the H–W model provide measures of the “strength” of reproductive isolation independent of the local frequency of the parental taxa, thereby allowing more meaningful comparisons of reproductive isolation across locales.

We estimated the parameters *s* and s* by fitting nonlinear weighted least‐squares regressions to the observed frequency of heterogamous mating or hybrids in a population, according to the equations *h *= 2*s* (*p *– *p* ^2^) or *h *= 2s* (*p* – *p* ^2^) using the function *nls* in R. We used different data sets to estimate *s* and s*. Estimates of premating isolation (*s*) and hybridization (s*) were calculated from the frequency of heterogamous insemination and prevalence of hybrids in either adult or larval populations from Burkina Faso and Cameroon, except for *An. arabiensis *× *An. gambiae s.s*. hybridization assessment. In the latter case, in fact, not a single *An. arabiensis* × *gambiae s.s*. hybrid was recorded in either Burkina Faso or Cameroon. To overcome this limitation, we estimated the parameter s* from published records of the prevalence of *An. arabiensis* × *gambiae s.s*. hybrids from East Africa (Table 1), where the confounding occurrence of *An. coluzzii* does not apply.

**Table 1:** Hybrids prevalence in larval and adult samples of *Anopheles gambiae s.l*. estimated by meta‐analysis. The analysis is based on a systematic review of 99 field studies retrieved from 94 articles published between 1964 and 2013 (see Appendix S2)

Geographical region	Life stage							
	Larvae				Adults			
	No. studies	Frequency (95% C.I.)	Cochran *Q* (*df*)	*I* ^2^ (95% C.I.)	No. studies	Frequency (95% C.I.)	Cochran *Q* (*df*)	*I* ^2^ (95% C.I.)
*Anopheles arabiensis *×* gambiae* (Giles)								
Africa	21	0.02% (0.01%–0.05%)	5.11^NS^ (19)	0% (0%–42%)	65	0.02% (0.02%–0.04%)	62.53^NS^ (59)	6% (0%–32%)
East Africa	11	0.02% (0.01%–0.06%)	1.91^NS^ (10)	0% (0%–51%)	22	0.03% (0.01%–0.07%)	12.83^NS^ (19)	0% (0%–42%)
West Africa	10	0.02% (0.00%–0.06%)	3.13^NS^ (8)	0% (0%–54%)	43	0.01% (0.00%–0.02%)	45.59^NS^ (37)	19% (0%–46%)
*Anopheles coluzzii *×* gambiae s.s*.								
West Africa	4	0.39% (0.16%–0.73%)	2.19^NS^ (3)	0% (0%–64%)	57	0.44% (0.18%–0.81%)	356.77*^**^ (39)	89% (86%–91%)
West Africa w/out HHA	4	0.39% (0.16%–0.73%)	2.19^NS^ (3)	0% (0%–64%)	50	0.17% (0.06%–0.33%)	112.57*^**^ (32)	72% (58%–79%)
West Africa w/out HHA and BF	4	0.39% (0.16%–0.73%)	2.19^NS^ (3)	0% (0%–64%)	37	0.07% (0.02%–0.14%)	27.37^NS^ (21)	23% (0%–54%)

HHA, high hybridization area, including coastal fringe of Guinea Bissau and Senegambia (estuary of the river Gambia and Casamance in Senegal); BF, Burkina Faso; NS: *p *> .05; ***: *p* < .001.

### 2.6 Ecological divergence among members of the *Anopheles gambiae* complex

One of the predictions of ecological speciation theory is that reproductive isolation should correlate directly with the extent of ecological divergence (Funk, Nosil, & Etges, 2006). We aimed to verify this prediction in our biological system by quantifying the degree of ecological divergence among the three species under study, and comparing it to the strength of pre‐ and postmating isolation assessed from previous analyses. To assess the degree of ecological divergence, we calculated bootstrapped Pianka indices of niche overlap with the R package *spaa* (Ulrich & Gotelli, 2010; Zhang, 2004). The Pianka index is a symmetrical index assuming values between 0 and 1, derived from the competition coefficients of the Lotka–Volterra equations (Pianka, 1974): $O_{jk}=\sum_{i=1}^{n}\left(p_{ji}\times p_{ki}\right)/\sqrt{\sum_{i=1}^{n}p_{ji}^{2}\times p_{ki}^{2}}$, where *p*_*ji*_ and *p*_*ki*_ are the proportions of resource *i* used by species *j* and *k*, respectively. A value of 0 suggests that the two species do not share resources (max ecological divergence), while 1 indicates complete overlap (no ecological divergence). Here, we assume that species use all resources proportionally to their relative frequency in each locality. Thus, *p*_*ji*_ and *p*_*ki*_ were estimated by the relative proportion of each species (*An. arabiensis*,* An. coluzzii*,* An. gambiae s.s*.) and their hybrids (*An. arabiensis* × *coluzzii*,* An. arabiensis* × *gambiae s.s*., *An. coluzzii* × *gambiae s.s*.) co‐occurring in areas of sympatry. This assumption is obviously an approximation; accordingly, the index is intended mostly as a proxy resuming the ecological niche of these species based on a global assessment of resource use. The data used for the calculation of the index come from the systematic literature review and original data from the surveys in Burkina Faso and Cameroon.

### 2.7 Assessment of hybrid viability penalty

Comparing the prevalence of hybrids in larval and adult samples can provide insights about mechanisms of postmating isolation. A decrease in hybrid prevalence would suggest that hybrids suffer from some viability penalty across these life stages. Conversely, similar prevalence of hybrids among larval and adult samples in the presence of postmating isolation (assessed independently) would suggest that other mechanisms are in place.

If the signal/noise ratio is sufficiently high, evidence for hybrid disadvantage can be obtained from results of the meta‐analyses. Because of large heterogeneities in the frequency of hybrids across locales (cf. Section 3), however, it is more appropriate to compare hybrid frequencies measured syntopically and synchronously for both larval and adult samples. We have found no such studies from the systematic literature review, but have original data from two independent surveys, one each in Burkina Faso and Cameroon.

In Burkina Faso, larvae and adult *An. gambiae s.l*. were collected in parallel during September–October 2000 from the village of Goundry, about 35 km north‐east of the capital Ouagadougou. Overall, 76 larval habitats and 89 human dwellings were sampled across an area of ~4 km^2^. In Cameroon, parallel larval and adult collections were carried out along an 18‐km urbanization gradient in Yaoundé. These surveys have already been presented (cf. Section 2.3), and further details are available in Kamdem et al. (2012).

## 3 RESULTS

We examined reproductive isolation among *An. coluzzii*,* An. gambiae s.s*. and *An. arabiensis* with a multipronged approach, taking as indicator of hybrid genotypes the heterozygous patterns of species‐specific diagnostic markers in the pericentric region of chromosome‐X (Fanello et al., 2003; Favia et al., 2001; Santolamazza et al., 2008). First, we performed a systematic review and meta‐analysis of published surveys reporting on hybrid prevalence, which overall showed strong reproductive isolation at a continental scale despite geographically restricted exceptions. Second, we exploited our own extensive field data sets collected at a regional scale in two contrasting environmental settings, that is in the arid savannah of Burkina Faso in West Africa, and in the moist rainforest of Cameroon in Central Africa, to analyse: (i) levels of premating isolation in the species triplet; (ii) the spatio/temporal and frequency‐dependent dynamics of hybridization, (iii) the relationship between postmating isolation and ecological divergence and (iv) hybrid viability penalty.

### 3.1 Hybrid prevalence at a continental scale

To assess the level of hybridization among *An. coluzzii*,* An. gambiae s.s*. and *An. arabiensis* in the African continent, we performed a systematic review of field studies reporting the occurrence and frequency of populations in sympatry that were published between 1964 and March 2013. Table 1 summarizes the results of the meta‐analyses based on 99 studies (retrieved from 94 published articles, Appendix S2) that satisfied the inclusion criteria, and whose ancillary data are collated in Tables S1–S4. The analyses pertaining to adult hybrids covered 25 countries with a total of ~135,000 and ~20,000 specimens identified to investigate rates of hybridization between *An. arabiensis* and *An. gambiae* (Giles), or *An. coluzzii* and *An. gambiae s.s*., respectively. Studies reporting the occurrence of larval hybrids were rarer: only six countries were covered, for a total number of identified specimens not exceeding 18,000. The distribution of specimens across countries was heterogeneous, with a few countries better covered than most. Despite these limitations and the sparseness of the data set, the analyses were based on a suitable number of adult hybrids: 17 and 160 for *An. arabiensis* × *gambiae* (Giles) and *An. coluzzii* × *gambiae s.s*., respectively. That was not the case for larval hybrids, with only four occurrence records of *An. coluzzii* × *gambiae s.s*., and no records for *An. arabiensis* × *gambiae* (Giles) (Tables S1–S4). It should be noted that in the case of larvae, estimates do not take into account the occurrence of male hybrids, which cannot be recognized due to hemizygosity of the diagnostic X‐linked rDNA markers. Thus, if we assume that both genders are equally represented in the population and sampling is unbiased, hybrids prevalence should be adjusted by a twofold increase in all larval samples.

The prevalence of *An. arabiensis* × *gambiae* (Giles) hybrids was homogeneous across studies regardless of life stage or major geographical region considered (for both adults and larvae, ~0.02% across the continent). On the other hand, frequency of hybrids observed between *An. coluzzii* and *An. gambiae s.s*. was approximately 20× higher (0.39% and 0.44% for larvae and adults, respectively) but not homogeneous across studies/sites (Table 1), as expected due to the known existence of a restricted “high hybridization area” (HHA) at the westernmost limit of their distribution range (Caputo et al., 2008, 2014; Marsden et al., 2011; Oliveira et al., 2008). It is important to note that in the core of HHA in Guinea Bissau, IGS‐heterozygous patterns cannot be taken as proxy of F1 hybrids as in the rest of Africa (Santolamazza et al., 2011). In fact, genomic studies revealed that these patterns result from high hybridization rates stable since at least the 1990s (Oliveira et al., 2008) leading to a novel “hybrid form” (Vicente et al., 2017) with polymorphic ribosomal IGS alleles due to recombination within chromosome‐X centromeric regions as well as within the multicopy ribosomal region (Caputo et al., 2016).

*Anopheles coluzzii* × *An. gambiae s.s*. hybrid prevalence is reduced to 0.17%—a value still 8× higher than the estimated prevalence of *An. arabiensis* × *gambiae* (Giles) hybrids—if the meta‐analysis is run excluding records from the HHA. While exclusion of the HHA data reduced the degree of heterogeneity between studies, the values of the Cochran *Q* and *I* ^2^ statistics still indicated the occurrence of significant between‐studies variation. The estimated prevalence of hybrids, however, conforms to a homogeneous distribution across studies and becomes 2.5× lower (0.07%) than the corresponding estimate for the full data set minus the HHA if records from Burkina Faso are also excluded (Table 1). This suggests that comprehensive sampling such as that carried out in Burkina Faso (where *An. coluzzii* × *gambiae s.s*. hybrids have been occasionally found at high frequency at a few locations (Costantini et al., 2009) is needed to provide a robust picture of the heterogeneity in *An. coluzzii *× *An. gambiae s.s*. hybridization where these species occur in sympatry.

### 3.2 Premating isolation at local scales

To investigate the strength of sexual isolation in the three species, we assessed the degree of pairwise assortative mating of *An. arabiensis*,* An. coluzzii* and *An. gambiae s.s*. by molecularly typing the sperm isolated from the spermatheca of females collected from three populations from central Burkina Faso, and a population from southern Cameroon.

In Burkina Faso, among 1,222 spermathecae that were isolated, 75 did not contain any sperm bundle, giving an average insemination rate of 93.9%. The remaining spermathecae were analysed, and 991 sperm bundles (representing 86.4% of the total sample) were molecularly identified. Homospecific insemination rates were 97.8% for *An. arabiensis*, 95.3% for *An. coluzzii* and 91.2% for *An. gambiae s.s*. (Table S5). Interestingly, one each of the three *An. arabiensis* × *coluzzii* hybrid females whose sperm content could be identified were mated, with a male of *An. arabiensis*,* An. coluzzii* or *An. gambiae s.s*., respectively, suggesting that hybrids copulate randomly; that is, hybridization breaks down premating isolation. In Cameroon, the analysis of 63 sperm bundles revealed rates of homospecific mating corresponding to 98% (53/54) in *An. gambiae s.s*., and 89% (8/9) in *An. coluzzii*, the difference being not significant (*G* test: χ^2^ = 1.50, *df* = 1, *p *= .221).

**Figure 2:**
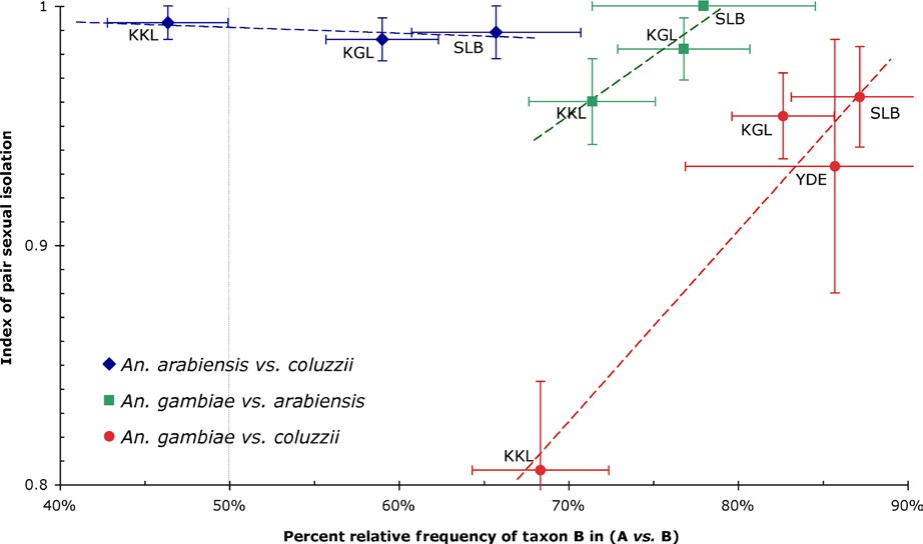
Indices of pair sexual isolation (*I*_PSI_ ± *SD*) among *Anopheles arabiensis*,* Anopheles coluzzii* and *Anopheles gambiae s.s*. Values of *I*_PSI_ for populations from Burkina Faso and Cameroon are plotted with respect to the estimated relative frequency (±2*SE*) of each taxon in the population; for example, the 50% value on the abscissa (vertical dotted line) represents equal frequencies of both focal taxa in the population. Fitted regression lines (dashed) are those returned by the minimal adequate generalized linear model No. 1 in Table S6. KGL, Kougoulapaka (Burkina Faso); KKL, Koukoulou (Burkina Faso); SLB, Salbisgo (Burkina Faso); YDE, Yaoundé (Cameroon)

As expected, all indices of pair sexual isolation (*I*_PSI_) significantly departed from random mating (*p *≪ .001), with values >0.8 and mostly close to complete assortative mating (i.e., *I*_PSI_ = 1; Figure 2). In most localities, premating isolation was stronger between *An. arabiensis* vs. *An. coluzzii* than between *An. arabiensis* vs. *An. gambiae s.s*., and much weaker between *An. coluzzii* vs. *An. gambiae s.s*. (Figure 2). Moreover, in one sampling site in Burkina Faso (Koukoulou) sexual isolation between *An. coluzzii* and *An. gambiae s.s*. was markedly lower than in other localities.

Results from generalized linear modelling provided statistical support to the full model (No. 1 in Table S6, containing all main effects, FREQUENCY and TAXA, as well as their interaction), indicating the occurrence of specific differences in frequency‐dependent sexual isolation as well as differences in the strength of reproductive isolation in this species triplet. The parameter estimates of the minimal adequate model and their statistical significance, as assessed by Student's *t* tests, are provided in Table 2. The slope of the regression lines for species pairs involving *An. arabiensis* was not significantly different from zero (Table 2), indicating that the premating barrier in this species did not conform to a frequency‐dependent mode of isolation as in the case of *An. coluzzii* and *An. gambiae s.s*. Assuming that the linear relationship is symmetrical across *p*_*ijk*_ = 0.5, predictions of the model provide estimates of the minimum expected degree of sexual isolation among this species pair at *p*_*ijk*_ = 0.5, which is *I*_PSI_ = 0.666.

**Table 2:** Parameter estimates of the minimal adequate generalized linear model quantifying the effect of frequency‐dependent hybridization among *Anopheles arabiensis* (species 1), *Anopheles coluzzii* (species 2) and *Anopheles gambiae s.s*. (species 3), based on the strength of their pair sexual isolation indices *I*_PSI_. The subscripts of the coefficients refer to the hybridizing species pair *i*,* j*. Results are presented according to the usual GLM output; that is, the intercept and slope of each species pair are expressed as the difference with respect to the first species pair (*i *= 1, *j *= 2, that is *An. arabiensis* vs. *An. coluzzii*)

Model terms	Parameter estimate	*SE*	*t*	*p*
Intercept (α_1,2_)	1.00317	0.06749		
Slope (β_1,2_)	−0.02427	0.11717	0.207	.846
Frequency: *An. arabiensis* vs. *An. gambiae s.s*. (∆β_1,3_)	0.52427	0.33523	1.564	.193
Frequency: *An. coluzzii* vs. *An. gambiae s.s*. (∆β_2,3_)	0.82214	0.16066	5.117	.007
Taxa: *An. arabiensis* vs. *An. gambiae s.s*. (∆α_1,3_)	−0.39917	0.24624	1.621	.180
Taxa: *An. coluzzii* vs. *An. gambiae s.s*. (∆α_2,3_)	−0.73569	0.11205	6.566	.003

### 3.3 Spatial and temporal dynamics of hybridization along a “contact zone” in Cameroon

To examine the impact of local factors upon hybridization at a microgeographical scale, we looked at the spatial and temporal distribution of larval and adult hybrids along an urbanization gradient in the capital of Cameroon—Yaoundé (Kamdem et al., 2012). Along this gradient, *An. coluzzii* is the sole species occurring in the core of the urban habitat, *An. gambiae s.s*. is the only species occurring in the rural habitat, while in a 6‐km‐wide “hybrid zone” in the middle of the gradient the two taxa are sympatric in a cline of relative frequencies. At the temporal scale, the prevalence of hybrids was serially correlated in time (Standardized Bartels Statistic = −2.29, RVN ratio = 0.678, *p* = .02): observed hybridization rates increased in July, peaked in August, to decline later on below the threshold of detection after October (Table S7). Spatially, hybrids occurred in localities 8‐7‐5‐4 of fig. 1E in Kamdem et al. (2012), in that temporal order, which is consistent with the “contact zone” of *An. coluzzii* and *An. gambiae s.s*. observed along the urbanization gradient (Kamdem et al., 2012). Considering the relative dynamics of the two populations, this sequence corresponds to the advancing front of the “contact zone” as the *An. coluzzii* population expands towards *An. gambiae s.s*. habitat, when the *An. gambiae s.s*. population density declines (fig. 2B in Kamdem et al., 2012). The occurrence of hybrids, therefore, was heterogeneous in space and time, and positively correlated with the degree of contact between the parental populations. This clearly highlights that, to obtain appropriate estimates of hybrid prevalence, we must consider how the frequency of the parental taxa in the population affects the prevalence of hybrids.

### 3.4 Frequency‐dependent hybridization at regional scales

The previous results have shown how hybridization varies at continental as well as microgeographical scales. From the systematic review, it emerges that most studies have focused on the analysis of adult mosquitoes, which is the life stage most closely associated with disease transmission and that the prevalence of hybrids between *An. coluzzii* and *An. gambiae s.s*. is highly heterogeneous at both geographical scales. To obtain meaningful estimates of hybrid prevalence, therefore, it is necessary to study in more detail the patterns and underlying causes of such heterogeneity. As a first step in this direction, we investigated the impact on hybridization of the relative frequency of the parental taxa in the population.

**Figure 3:**
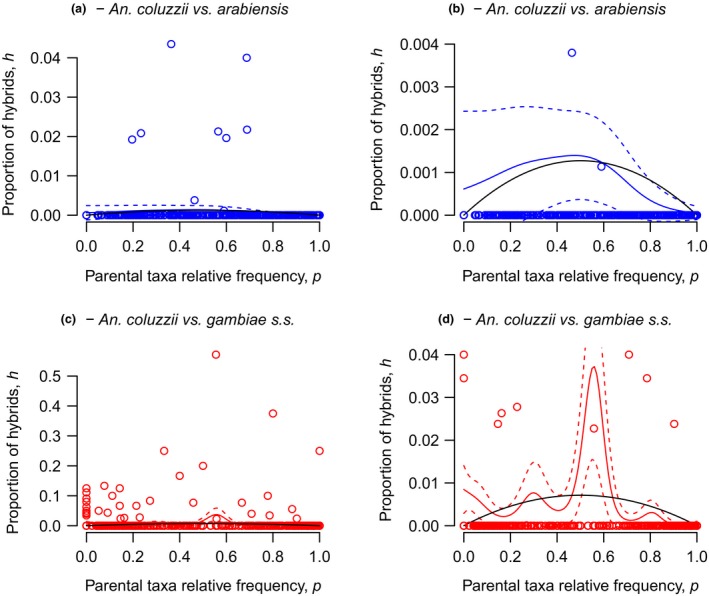
Frequency‐dependent hybridization in Burkina Faso. Observed prevalence of adult hybrids (*ĥ*) plotted against the estimated relative frequency of the parental taxa in the population (*p̂*), with each dot representing a sample from a single locality. (a, b) *Anopheles coluzzii *× *arabiensis*; (c, d) *Anopheles coluzzii* × *gambiae*. Panels (b) and (d) show the same data as panels (a) and (c), respectively, with the ordinate cropped for visualization purposes. The continuous coloured curves in the four panels (±2*SE*, dotted coloured curves) represent the predicted values of *h* from the GAM models including a nonparametric smoothed function of *p*. The black inverted parabolas depict the theoretical expected frequency of hybrids from Hardy–Weinberg expectations when only a fraction of individuals in the populations mate at random and a fraction s*/*s* of heterogamous zygotes survive

**Figure 4:**
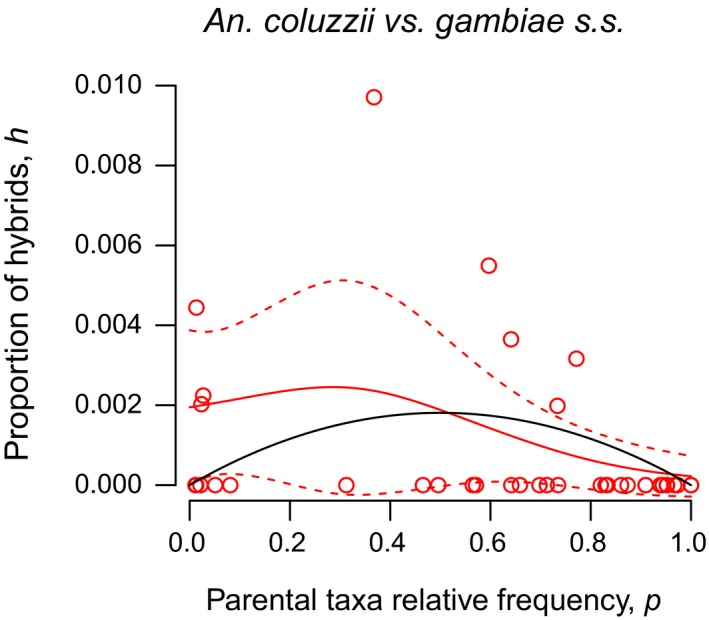
Frequency‐dependent hybridization in Cameroon. Observed prevalence of *Anopheles coluzzii* × *gambiae* larval hybrids (*h*) plotted against the estimated relative frequency of the parental taxa in the population (*p*). For an explanation of symbols, refer to Figure 3

Despite noisy data sets, in all cases the nonparametric smoothers of the GAM analysis extracted a curvilinear relationship between the prevalence of adult or larval hybrids and the frequency of the parental taxa, with maxima at intermediate frequencies and local minima at the extremes of the range of frequencies (Figures 3 and 4). Accordingly, it can be inferred that the expected prevalence of hybrids in a population is highest when the parental taxa occur at comparable frequencies, that is when their degree of contact is maximal. In all instances, the nonparametric smoothers accounted for a significant proportion of the deviance explained, which was comparable across species pairs, and models including the smoothers showed the lower AIC compared to the null model with only the parametric term α (Table 3). These results justify the H–W approach for assessment of postmating isolation from these data sets, as detailed below (Section 3.6).

**Table 3:** Results of generalized additive models fitted to the frequency of hybrids in field samples from Burkina Faso and Cameroon. The models test the significance of nonparametric smoothed functions of the explanatory variable “relative frequency of the parental taxa” *p* on the response variable “frequency of hybrids” *h* (cf. Figures 3 and 4). Statistical significance in each case was assessed by likelihood ratio tests (*D*) and by the difference in the Akaike information criterion (∆AIC) among the model containing the nonparametric smoothed function and the null model without the smoother. Negative values of ∆AIC denote that the AIC of the model with the smoother was lower than that of the model without the smoother. *n*: number of samples; *edf*: equivalent degrees of freedom; χ^2^: chi‐square value; *p*:* p*‐value

Hybridizing species	*n*	Model statistics				Nonparametric smoother			Parametric parameter (α)	
		∆AIC	*D*	*p*	Explained deviance	*edf*	χ^2^	*p*	Estimate	*p*
*An. arabiensis *×* coluzzii*—Burkina Faso (adults)	558	−4.48	9.33	.001	13.9%	2.374	4.4	.224	−7.5575 (±0.4467)	<.001
*An. coluzzii *×* gambiae s.s*.—Burkina Faso (adults)	558	−62.67	79.82	<.001	19.8%	8.557	60.9	<.0001	−6.2725 (±0.2622)	<.001
*An. coluzzii *×* gambiae s.s*.—Cameroon (larvae)	38	−1.71	5.08	.01	22.9%	1.686	3.4	.194	−7.0731 (±0.4368)	<.001

### 3.5 Postmating isolation at local scales

From the observed rates of homospecific and heterospecific insemination, it is possible to estimate the expected frequency of individuals belonging to each taxon and their hybrids in the absence of postmating isolation. We carried out such calculations for the three populations in Burkina Faso where we measured homospecific and heterospecific mating frequencies. The observed number of mosquitoes belonging to each taxon departed significantly from expectation in each locality (*G* test = 243.1; *df* = 5; *p* < .01) as well as on the aggregated data across localities (chi‐square goodness‐of‐fit test = 137.36, *df*= 5; *p *< .0001), showing a strong deficit of hybrids and excess of parental taxa (Table 4).

**Table 4:** Estimated frequency of *Anopheles gambiae s.l*. taxa based on rates of homospecific and heterospecific mating in three populations from Burkina Faso

Locality	*An. arabiensis* (A)		*An*. *coluzzii* (C)		*An. gambiae s.s*. (G)		A* *×* *C		A* *×* *G		C* *×* *G	
	Obs.	Exp.	Obs.	Exp.	Obs.	Exp.	Obs.	Exp.	Obs.	Exp.	Obs.	Exp.
Kougoulapaka	361	355.3	520	504.4	109	105.3	1	5.7	0	2.5	0	16.8
Koukoulou	422	413.5	365	331.1	169	148.5	3	4.0	0	10.7	0	51.6
Salbisgo	124	124.0	238	236.1	397	355.9	0	1.9	0	0.0	0	41.1
Total	907	892.8	1123	1071.6	675	609.7	4	11.6	0	13.2	0	109.5

Similarly, the analysis of *An. coluzzii* hybridization with *An. gambiae s.s*. in southern Cameroon showed that the frequency of heterospecific mating was significantly greater than the prevalence of larval hybrids (Pearson's chi‐square test with Yates’ continuity correction: χ^2^ = 11.9; *df* = 1; *p *< .001), even more so in the case of adult samples, given that not a single *An. coluzzii* × *gambiae s.s*. hybrid was observed out of 1,465 adult mosquitoes of the two species collected in the area (Kamdem et al., 2012).

These results suggest that postmating isolation contributes to the reproductive barrier among these taxa. However, the evaluation of the strength of postmating isolation relative to the premating barrier is hampered by the large sampling error associated with results based on surveys from single localities and on relatively small samples (e.g., in Burkina Faso, only four hybrids were observed despite >2,700 mosquitoes collected and analysed, see Table 4), as well as by the dependence of hybridization on the frequency of the parental species, as demonstrated from previous analyses. Therefore, to provide an average estimate of the strength of postmating isolation relative to premating isolation, we have also implemented the alternative analytical approach represented by the H–W model, as detailed below.

### 3.6 Postmating isolation and ecological divergence at regional scales

Estimates of the *s* and s* parameters of the H–W model are presented in Table 5. The strength of premating isolation quantified by the parameter *s* of the H–W model conformed to the expectation of direct proportionality from time since lineage divergence: isolation was weakest between the most recently diverged species, that is *An. coluzzii* and *An. gambiae s.s*., and strongest between the phylogenetically most distant, that is *An. arabiensis*–*An. gambiae s.s*. and *An. arabiensis*–*An*. *coluzzii* (Table 5). A similar pattern resulted for the parameter s* (Table 5); however what is of more interest is the relative contribution of postmating isolation expressed by the ratio s*/*s*. In this case, the pattern was reversed and associated more closely with the strength of ecological divergence (Table 5): the species pairs with stronger postmating isolation were those showing stronger ecological divergence, that is *An. arabiensis* vs. *An. gambiae s.s*. and *An. coluzzii* vs. *An. gambiae s.s*. in Cameroon (Pianka's index, 0.562 ± 0.098 and 0.317 ± 0.123, respectively) and that with weakest postmating isolation was that with greatest ecological overlap, that is *An. arabiensis* and *An. coluzzii* in Burkina Faso (Pianka's index, 0.717 ± 0.111). Notably, assessment of ecological niche overlap between *An. coluzzii* × *gambiae s.s*. hybrids and the parental species showed (Table 6) that hybrids co‐occurred significantly more with *An. gambiae s.s*. than *An. coluzzii* in both Burkina Faso (Pianka's index 0.107 ± 0.039 vs. 0.025 ± 0.009, respectively, in adults) and Cameroon (Pianka's index 0.715 ± 0.113 vs. 0.321 ± 0.108, respectively, in larvae).

**Table 5:** Assessment of the relative contribution of pre‐ versus postmating isolation in relation to ecological divergence according to the H–W model. Estimates (±*SE*) of premating isolation and hybridization are based on the frequency of heterogamous insemination and prevalence of hybrids in either adult or larval populations from Burkina Faso and Cameroon, except for *Anopheles arabiensis *×* Anopheles gambiae s.s*. hybridization assessment that is based on populations from East Africa. The parameter *m* estimates the proportion of nonassortative matings; parameters *s* and s* estimate the proportional decrease in hybrids from H–W expectations due to assortative mating (*s*) and total reproductive isolation (s*). The parameter s*/*s* estimates the proportion of heterogamous inseminations producing viable hybrids in the population, which is a measure of the strength of postmating isolation relative to premating isolation Symbols +, ++, +++, ++++ represent ranks of incresing strength of each parameter

Hybridizing species (Life Stage, Country)	Proportion mating assortatively (1–*m*) %	Premating isolation (*s*)	Hybridization (s*)	Postmating isolation (s*/*s*) %	Ecological Niche overlap (Pianka's *O*_*jk*_)	Inferred strength of premating isolation	Inferred strength of postmating isolation	Inferred strength of ecological divergence
*An. arabiensis *×* An. coluzzii* (Adults)	89.3	0.01139 (±0.0018)	0.0025475 (±0.0004491)	22.4	0.717 (±0.111)	+++	+	+
*An. arabiensis *×* An. gambiae s.s*. (Adults)	83.5	0.02722 (±0.0082)	0.0006083 (±0.0002451)	2.2	0.562 (±0.098)	++	++++	++
*An. coluzzii *×* An. gambiae s.s*. (Adults, BF)	65.4	0.11967 (±0.0400)	0.014258 (±0.004303)	11.9	0.492 (±0.097)	+	++	+++
*An. coluzzii *×* An. gambiae s.s*. (Larvae, CM)	65.4	0.11967 (±0.0400)	0.007238^a^ (±0.001099)	6.0	0.317 (±0.123)	+	+++	++++

BF, Burkina Faso; CM, Cameroon.

a Value doubled because of hemizygosity of males in the larval sample.

**Table 6:** Pianka indices of niche overlap (±*SD*) between hybrids and parental taxa

Species	*An. arabiensis *×* coluzzii*	*An. coluzzii *×* gambiae s.s*. (BF, adults)	*An. coluzzii *×* gambiae s.s*. (CM, larvae)
*An. arabiensis*	0.572 ± 0.207	0.047 ± 0.024	—
*An. coluzzii*	0.391 ± 0.165	0.025 ± 0.009	0.321 ± 0.108
*An. gambiae s.s*.	0.297 ± 0.173	0.107 ± 0.039	0.715 ± 0.113

—, Not calculable because of *An. arabiensis* absence; BF, Burkina Faso; CM, Cameroon.

### 3.7 Hybrid viability penalty

Evidence for hybrid disadvantage was found from results of the meta‐analysis for *An. coluzzii* × *gambiae s.s*. hybrids, but not for *An. arabiensis* × *gambiae s.s*. (Giles) hybrids (Table 7). From the syntopic and synchronous surveys carried out in Burkina Faso and Cameroon, the prevalence of *An. coluzzii* × *gambiae s.s*. hybrids was on average approximately fivefold higher in larval compared to adult samples (Table 7). Evidence for selection against hybrids, however, was not statistically significant in each individual study and only close to the 5% threshold of significance in the combined analysis. In the case of *An. arabiensis* × *gambiae* (Giles) hybrids, prevalence estimates in larval and adult samples from Burkina Faso were close to each other, suggesting that these hybrids do not suffer a detectable survival disadvantage.

**Table 7:** Assessment of viability penalty in hybrids. Percent frequency of hybrids in concurrent larval and adult samples. Frequencies of larval hybrids were doubled to take into account the nondetectability of hybrid males due to hemizygosity of the diagnostic marker (for further details, see text)

Country/locality	Larvae (*n*)	Adults (*n*)	*G* test	*p*	Source
*An. arabiensis *×* gambiae* (Giles)					
Africa	0.04%	0.02%			Meta‐analysis, Table 1
Burkina Faso/Goundry	0.07% (1,382)	0.09% (1,172)	0.01 (*df* = 1)	.920	Yr. 2000 survey, Appendix S1
*An. coluzzii *×* gambiae s.s*.					
West Africa	0.78%	0.07%			Meta‐analysis, Table 1
Burkina Faso/Goundry	0.50% (796)	0.14% (739)	1.70 (*df* = 1)	.192	Yr. 2000 survey, Appendix S1
Cameroon/Yaoundé	0.63% (1,276)	0.00% (114)	1.36 (*df* = 1)	.244	Yaoundé survey, Table S7
Burkina Faso + Cameroon total	0.58% (2072)	0.12% (853)	3.67 (*df* = 1)	.055	

## 4 DISCUSSION

In this work, we provide a view of the emergence and modulation of reproductive isolation among three sympatric members of the *An. gambiae* species complex by means of independent measures of the strength of premating versus postmating isolation that account for the sequential nature of the reproductive barrier.

First, we have performed a systematic review and meta‐analysis of published surveys reporting on hybrids prevalence, which has also served to evaluate the variability and strength of reproductive isolation at a continental scale. Then, we have analysed our own field data sets collected at a regional scale in two contrasting environmental settings, that is in the arid savannah of Burkina Faso in West Africa, and in the rainforest of Cameroon in Central Africa. In this way, we have gathered the most exhaustive data set to date of hybrids occurrence and frequency in this species triplet, which has facilitated comparisons of the expected prevalence of hybrids in locales where we also obtained field measures of interspecific mating events. The emerging pattern from these results is that in this species triplet sexual isolation is positively correlated with phylogenetic distance. However, in the case of postmating isolation, we have found evidence for the occurrence of ecologically based modulation of the strength of the reproductive barrier. Accordingly, we have also looked for evidence of selection against hybridization by comparing the prevalence of hybrids in larval and adult samples, with the expectation that in the presence of selection against hybrids, the prevalence should decrease across stages as a consequence of hybrid mortality.

The main conclusions are further discussed below. It must be underlined, however, that rare events are challenging to study, and despite the analysis of several tens of thousands mosquitoes and an extensive data set gathered during more than 10 years of field studies across Africa, our results are still based only on some tens to a few hundreds of hybrid individuals. Moreover, the identification of hybrids was based on typing only the species‐diagnostic X‐linked rDNA locus, which cannot provide information about advanced generation hybrids and levels of population admixture. These limitations should be kept in mind when gauging the level of confidence we can attach to these results. On the other hand, our data set is the most comprehensive to date to allow such kind of analyses.

### 4.1 Ecological postmating isolation

Support for the occurrence of extrinsic postmating isolation in this species trio has emerged from two pieces of evidence. On the one hand, the observed frequency of hybrids in populations at both local and regional scales was less than that expected from the observed frequency of heterogamous mating among these species. Significant deficiencies of hybrids were demonstrated both in adult populations from Burkina Faso and in larval populations from Cameroon. On the other hand, as expected when barriers to gene flow evolve as a result of ecologically based divergent selection (Rundle & Nosil, 2005; Schluter & Conte, 2009), the strength of reproductive isolation was proportional to the degree of ecological divergence, with postmating isolation being weaker between the more ecologically similar taxa (i.e., *An. arabiensis* and *An. coluzzii*), compared to the species pairs characterized by lower ecological overlap. It should be noted, however, that we defined the degree of ecological divergence only by habitat overlap, as assessed by co‐occurrence in each locale, while more comprehensive assessments of ecological niche partitioning (e.g., McBride & Singer, 2010) are needed to strengthen these conclusions.

Another caveat concerns the frequency in each population of backcross F*n* hybrids, which are undetectable by our identification diagnostics. If this frequency is biased in favour of *An. coluzzii *× *An. gambiae s.s*. relative to those hybrids involving *An. arabiensis* as one of the parental species (due to some degree of infertility of backcrosses with *An. arabiensis* [Slotman et al., 2004]), we may be underestimating postmating isolation in *An. coluzzii* vs. *An. gambiae s.s*. relative to intercrosses involving *An. arabiensis* because F1 hybrids may be overestimated in the former case. Nevertheless, it should be noted that this bias is probably minor considering the relative rarity of hybridization in this species triplet overall. Moreover, underestimating the strength of postmating isolation in crosses involving *An. coluzzii* and *An. gambiae s.s*. does not perturb the validity of the above conclusions, and quite the reverse would strengthen them.

Available data are insufficient to elucidate the nature of extrinsic postmating selection, as there is weak statistical support for some degree of viability penalty only in the case of *An. coluzzii *× *gambiae s.s*. hybrids. Unless larger samples are obtained or focussed studies are carried out, statistical inference about rare events such as hybridization in this species complex remains challenging. Moreover, ecological postzygotic isolation might be expressed also early on in hybrid embryos, or during the early stages posteclosion, and therefore be undetectable by the comparative method implemented in this study. Some behavioural and physiological differences between larval *An. coluzzii* and *An. gambiae s.s*. associated with potential selective forces modulating habitat segregation have been uncovered (Dao et al., 2014; Tene Fossog et al., 2014; Gimonneau et al., 2012; Lehmann & Diabate, 2008). However, much still needs to be done to identify the factors causing divergent selection in geographical populations living in different ecological contexts. Moreover, sexual selection mechanisms like cryptic prezygotic isolation might contribute in variable degrees to the strength of the reproductive barrier. Postinsemination cryptic reproductive isolation before fertilization might include directional or nondirectional cryptic female choice, and intersexual evolutionary conflict (Birkhead & Pizzari, 2002; Price, Kim, Gronlund, & Coyne, 2001). However, it has been suggested that both sexual conflict and many forms of sexual selection are expected to operate independently from the environment, so that other forms of cryptic postmating isolation mechanisms that occur after copulation but before hybrid offspring are produced, like reduced female fitness resulting from heterogamous insemination, better explain those instances in which taxa exhibit stronger reproductive isolation in ecologically divergent populations (Nosil & Crespi, 2006).

### 4.2 Frequency‐dependent hybridization

Overall, we found that higher prevalence of hybrids in natural field populations was associated with locales where the relative abundance of the parental taxa was closer to 1:1 proportions. This frequency‐dependent pattern of hybrid prevalence is concordant with the frequency‐dependent pattern of sexual isolation observed when considering *An. gambiae s.s*. as one of the parental taxa. The index of sexual isolation decreased as the relative abundance of this species in the population was closer to 50% relative to *An. coluzzii* or *An. arabiensis*. In this case, therefore, a higher prevalence of hybrids in the population is expected at intermediate frequencies of the parental taxa because under these circumstances premating isolation is also weaker. In the case of *An. arabiensis* and *An. coluzzii*, there was no evidence of frequency‐dependent sexual isolation, although in this case it is statistically more difficult to demonstrate such an effect, given that the strength of premating isolation between these species remained >97% regardless of the relative abundance of the parental taxa in the population.

Beyond the effect due to sexual isolation, the higher prevalence of hybrids at intermediate frequencies of the parental species is also compatible with the greater suitability of local environmental conditions for this class of individuals where neither of the parental taxa predominates. According to ecological speciation theory, the strength of extrinsic postmating selection against hybrids should be greater where the fitness of the parental species is greater, such as at the opposing ends of an ecological gradient to which each parental populations is specifically adapted (Kirkpatrick, 2001; Seehausen, 2004; Stankowski, 2013). At the ends of the gradient, therefore, each species will alternatively predominate over the other. It is in the centre of the gradient defining a hybrid zone with intermediate parental frequencies that the fitness of hybrids is expected to be greater, that is where environmental conditions are not optimal for either one of the parental taxa. This is consistent with the pattern of occurrence of hybrids observed along the urbanization gradient in Yaoundé, Cameroon: hybrids were only found in the centre of the gradient, which defines the contact zone between *An. gambiae s.s*. (largely predominating at the rural side of the gradient) and *An. coluzzii* (largely predominating at the urban side of the gradient).

In a few localities, *An. coluzzii *× *gambiae s.s*. hybrids were observed despite that only *An. gambiae s.s*. was collected. This finding can be interpreted in two, not mutually exclusive ways. On the one hand, when there is random mating (at least in a fraction of the population) and one of the two hybridizing taxa is rare relative to the other, it is expected that the relative frequency of hybrids is higher than that of the less represented species as a simple consequence of the Hardy–Weinberg law (these taxa have been identified by a single X‐linked SNP), and the observed pattern may simply reflect the effect of sampling (binomial) errors around the fitted model curve. The same pattern has also been observed in the HHA (Marsden et al., 2011), and it was interpreted as asymmetric mating events in which rare females from *An. coluzzii* mate with *An. gambiae s.s*. more easily because no *An. coluzzii* males are to be found. There is also another observation from our data set that is compatible with this explanation: hybrids co‐occur more with *An. gambiae s.s*. than *An. coluzzii* (Table 6), which may suggest that postmating isolation (or hybrids dispersal) is asymmetrical (i.e., stronger when *An. coluzzii* is more abundant). Unfortunately, our data set is probably too noisy to test this hypothesis.

### 4.3 Geographical mosaic of reproductive isolation and narrow bands of hybridization

The local (regional) pattern of hybrid prevalence described above appears embedded within continent‐wide differences in hybridization rates. We have quantitatively confirmed the occurrence of geographical heterogeneities between *An. coluzzii* and *An. gambiae s.s*. consistent with the “geographical mosaic of reproductive isolation” scenario highlighted by Lee et al. (2013), which is likely the product of the complex interactions among several—mostly unknown—factors. These mosaics can become apparent when data from areas sampled exhaustively are available, such as the case of Burkina Faso (this work) or Mali (Lee et al., 2013).

On the one hand, at a regional‐scale hybridization appears greater at intermediate frequencies of the parental taxa. On the other hand, local‐scale variability in hybridization rates might result from the history of geographical contact between diverging populations that have evolved at times in allopatry. There is chromosomal (Coluzzi, Sabatini, della Torre, Di Deco, & Petrarca, 2002; Simard et al., 2009), molecular (Lee et al., 2009; Slotman et al., 2006) and biogeographical (Fossog et al., 2014; Pinto et al., 2013) evidence of population structure within *An. coluzzii*, which may be distinguished in two, mostly allopatric, geographical “races,” one inhabiting in the xeric savannah of West Africa, and another one localized on the coastal fringe of the Gulf of Guinea (Tene Fossog et al., 2014). In Cameroon, for instance, we observed a higher hybridization rate in localities situated along the Cameroon Volcanic Line compared to neighbouring localities situated in the same ecoclimatic domain. These areas of higher than average hybridization may constitute regions of secondary contact between populations that have not “co‐evolved” together, that is coastal populations of *An. coluzzii* coming in contact with inland populations of *An. gambiae s.s*. Fine‐scale, not sparse, phylogeographic records are now crucially needed to test this hypothesis. According to present results, however, it is perhaps more appropriate to espouse the view that hybridization in this complex occurs mostly along narrow bands of primary or secondary contact between populations with divergent ecological requirements and geographical histories. Tene Fossog et al. (2014) have provided a predictive map of the relative abundance of *An. gambiae s.s*. and *An. coluzzii* in their range of sympatry. This map provides a template to construct expectations regarding the geographical distribution of *An. coluzzii* × *gambiae s.s*. hybrids: their prevalence is expected to be maximal along narrow bands where the two parental taxa are predicted to occur at comparable frequencies (areas in yellow‐light green in the map of their Figure 3). The rate of hybridization appeared to vary as a function of the degree of contact among taxa not only spatially, but also temporally. In the rain forest of Cameroon, for example, the degree of contact between *An. coluzzii* and *An. gambiae s.s*. weakens when *An. gambiae s.s*. populations collapse during drier periods due to lower availability of suitable rain‐dependent larval habitats (Tene Fossog et al., 2014; Kamdem et al., 2012). In the xeric savannah of West Africa, *An. gambiae s.s*. local populations extinguish during the dry season probably migrating towards more humid habitats (Dao et al., 2014) while *An. coluzzii* continues to breed in more permanent anthropogenic larval habitats like irrigated fields, artificial water reservoirs or rice paddies (Gimonneau et al., 2012).

At a continental scale, it is possible to hypothesize that also the HHA in Guinea Bissau and Senegambia has originated from secondary contact between populations that have evolved in allopatry (Caputo et al., 2014; Marsden et al., 2011; Pinto et al., 2013). Marsden et al. (2011) reported that hybridization between *An. coluzzii* and *An. gambiae s.s*. in Guinea Bissau led to asymmetric introgression, with gene flow occurring prevalently from *An. coluzzii* into the genome of *An. gambiae s.s*. These authors proposed that asymmetry was associated with populations where *An. gambiae s.s*. was the more prevalent taxon of the two: under these circumstances, if hybridization breaks down the isolation barrier, hybrids would mate nonassortatively and therefore would backcross mostly with the more abundant taxon. The swarming behaviour of *An. coluzzii* and *An*. *gambiae s.s*. in Burkina Faso supports the hypothesis that *An. gambiae* s.s females might be more prone to cross‐mating than *An. coluzzii* (Dabire et al., 2013), in particular when occurring in less favourable ecological contexts (Niang et al., 2015). This explanation is compatible with several outcomes of our study. First, it is significant, although admittedly conjectural because of the tiny sample, that we observed three *An. arabiensis* × *coluzzii* hybrids mating in the most nonassortative way possible, that is with one each of the three parental taxa available in the local population. This observation suggests that mate choice may be disrupted by hybridization. Second, we found that in our population from Burkina Faso *An. gambiae s.s*. was the least homogamous of the three taxa, suggesting that this species may be characterized by some mate choice traits leading it to behave more promiscuously than *An. arabiensis* or *An. coluzzii*. Notably, whole‐genome sequencing revealed that Guinea Bissau coastal region harbours a hybrid form characterized by an *A. gambiae*‐like sex chromosome and massive introgression of *A. coluzzii* autosomal alleles (Vicente et al., 2017).

## 5 CONCLUSIONS

**Figure 5:**
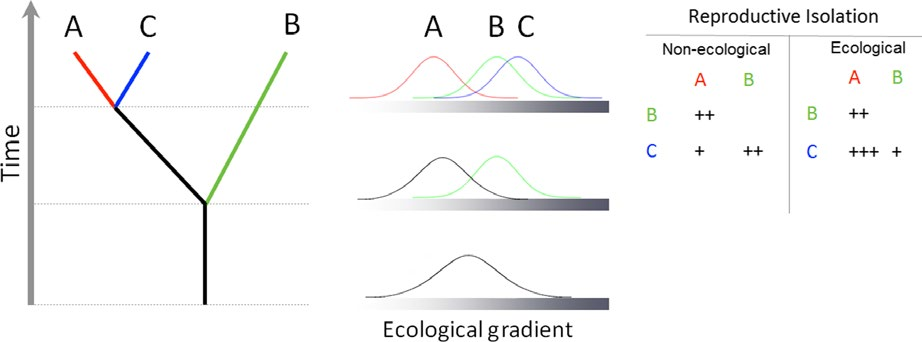
Theoretical framework outlining the expected relationships among time since lineage divergence, ecological divergence and the strength of reproductive isolation (+ < ++ < +++)

Investigating incompletely reproductively isolated taxa of the *Anopheles gambiae* complex, we have provided evidence that pre‐ and postmating reproductive barriers can follow different pathways in recent adaptive radiations. On the one hand, degree of premating isolation has been shown to increase in the course of lineage divergence, likely reflecting the accumulation of different species recognition mechanisms, independently of ecological context. On the other hand, postmating isolation appears to be modulated by the strength of current ecological divergence. This pattern is consistent with the scenario depicted in Figure 5 in which a group of three taxa with permeable reproductive barriers (A, B and C, corresponding in this case to *An. gambiae s.s*., *An. arabiensis* and *An. coluzzii*, respectively) show the degree of reproductive isolation that correlates with ecological divergence, and in which a taxon C is ecologically closer to the most phylogenetically distant taxon B. Under a mode of accumulation of barriers to gene flow that is independent of ecological processes, it is expected that isolation will follow the usual pattern of increase with time since lineage divergence. Conversely, when ecological factors foster the emergence and development of reproductive isolation, ecological speciation theory predicts that the taxa A and C are expected to be less isolated than A and B or B and C (Noor, 1997; Rundle & Nosil, 2005). In accordance with this scenario, we found that postmating barriers are stronger between *An. coluzzii* and *An. gambiae s.s*., and *An. arabiensis* and *An. gambiae s.s*., and weaker between *An. arabiensis* and *An. coluzzii* suggesting that ancestral postmating barriers can increase or be relaxed in response to ecological niche dynamics. These patterns appear to be modulated by the probability of interspecific encounters of the three taxa at a local scale, whereas at a continental scale hybridization seems to occur mostly along narrow contact areas between populations with divergent ecological requirements and geographical histories, demonstrating the unappreciated importance of ecogeographical isolation barriers in this species complex.

Incontrovertible evidence of naturally occurring F1 hybrids between species in the *An. gambiae* complex was first discovered half a century ago (White, 1971), and low levels of contemporary hybridization continue to be observed in surveys across sub‐Saharan Africa, as shown by the systematic review here presented (Table 1, Appendix S2 and Tables S1–S4). Until relatively recently, two factors hindered appreciation of the role of hybridization in this group. First, the influence of Ernst Mayr and Theodosius Dobzhansky perpetuated the prevailing view that hybrids were aberrant, evolutionary dead ends (Coyne & Orr, 2004; Seehausen, 2013). Second, the absence of genomic tools and resources precluded definitive demonstration of pervasive introgression. With the growing availability of whole‐genome sequences and powerful statistical approaches, and evidence of introgression uncovered even in our own genomes (Racimo, Sankararaman, Nielsen, & Huerta‐Sánchez, 2015), it is now becoming accepted that hybrids are important conduits of genetic information between parental species, without necessarily causing their collapse. Indeed, introgression has had a massive impact on the extent of shared genotypes, even among nonsister species (and important malaria vectors) in the *An. gambiae* complex (Bottà, 2017; Clarkson et al., 2014; Fontaine et al., 2015; The Anopheles gambiae 1000 Genomes Consortium, 2016).

Importantly, as our work and others have begun to reveal, while the occurrence of hybridization—on average—is relatively rare, the hybridization rate can wax and wane according to local ecological conditions. Future efforts should be devoted to an improved understanding of the conditions favourable to hybridization, as better predictions of when and where it occurs can inform models of the spread of insecticide or parasite resistance, as well as gene drive. Moreover, in much the same way understanding the rates and outcomes of hybridization has been important for risk assessment and the regulation of genetically engineered crops (Ridley & Alexander, 2016), defining the spatial and temporal dynamics of gene flow across species and populations of malaria vectors can help setting policies and regulations, and assist decision‐makers when considering mosquito genetic control technologies.

## CONFLICT OF INTEREST

The authors declare that they have no conflict of interest.

## DATA ARCHIVING STATEMENT

Data available from the Dryad Digital Repository: https://doi.org/10.5061/dryad.58k34.

## References

[eva12517-bib-0001] Bartels, R. (1982). The rank version of von Neumann's ratio test for randomness. Journal of the American Statistical Association, 77(377), 40–46.

[eva12517-bib-0002] Birkhead, T. R. , & Pizzari, T. (2002). Postcopulatory sexual selection. Nature Reviews, 3(4), 262–273. 10.1038/nrg774 11967551

[eva12517-bib-0003] Bottà, G. (2017). Evolutionary genomics of Anopheles gambiae and An. coluzzii. Rome, Italy: “Sapienza” Università di Roma.

[eva12517-bib-0004] Caputo, B. , Nwakanma, D. , Caputo, F. P. , Jawara, M. , Oriero, E. C. , Hamid‐Adiamoh, M. , … Della Torre, A. (2014). Prominent intraspecific genetic divergence within *Anopheles gambiae* sibling species triggered by habitat discontinuities across a riverine landscape. Molecular Ecology, 23(18), 4574–4589. 25040079 10.1111/mec.12866

[eva12517-bib-0005] Caputo, B. , Nwakanma, D. , Jawara, M. , Adiamoh, M. , Dia, I. , Konate, L. , … della Torre, A. (2008). *Anopheles gambiae* complex along The Gambia river, with particular reference to the molecular forms of *An. gambiae s.s* . Malaria Journal, 7, 182. 18803885 10.1186/1475-2875-7-182 PMC2569043

[eva12517-bib-0006] Caputo, B. , Pichler, V. , Mancini, E. , Pombi, M. , Vicente, J. L. , Dinis, J. , … Weetman, D. (2016). The last bastion? X chromosome genotyping of *Anopheles gambiae* species pair males from a hybrid zone reveals complex recombination within the major candidate “genomic island of speciation”. Molecular Ecology, 25(22), 5719–5731. 27661465 10.1111/mec.13840

[eva12517-bib-0007] Caputo, B. , Santolamazza, F. , Vicente, J. L. , Nwakanma, D. C. , Jawara, M. , Palsson, K. , … della Torre, A. (2011). The “far‐west” of *Anopheles gambiae* molecular forms. PLoS ONE, 6(2), e16415. 21347223 10.1371/journal.pone.0016415 PMC3039643

[eva12517-bib-0008] Carvajal‐Rodriguez, A. , & Rolan‐Alvarez, E. (2006). JMATING: A software for the analysis of sexual selection and sexual isolation effects from mating frequency data. BMC Evolutionary Biology, 6, 40. 16684352 10.1186/1471-2148-6-40 PMC1479385

[eva12517-bib-0009] Clarkson, C. S. , Weetman, D. , Essandoh, J. , Yawson, A. E. , Maslen, G. , Manske, M. , … Donnelly, M. J. (2014). Adaptive introgression between *Anopheles* sibling species eliminates a major genomic island but not reproductive isolation. Nature Communications, 5(May), 4248. 10.1038/ncomms5248 PMC4086683 24963649

[eva12517-bib-0010] Clements, A. N. (1999). The biology of mosquitoes: Sensory, reception, and behaviour (1st ed.). New York, NY: CABI.

[eva12517-bib-0011] Coetzee, M. , Craig, M. , & Le Sueur, D. (2000). Distribution of African malaria mosquitoes belonging to the *Anopheles gambiae* complex. Parasitology Today, 16(2), 74–77. 10652493 10.1016/s0169-4758(99)01563-x

[eva12517-bib-0012] Coetzee, M. , Wilkerson, R. , della Torre, A. , Coulibaly, M. B. , & Besansky, N. J. (2013). *Anopheles coluzzii* and *Anopheles amharicus*, new members of the *Anopheles gambiae* complex. Zootaxa, 3619(3), 246–274. 26131476

[eva12517-bib-0013] Coluzzi, M. , & Sabatini, A. (1967). Cytogenetic observations on species A and B of the *Anopheles gambiae* complex. Parassitologia, 9, 73–88.

[eva12517-bib-0014] Coluzzi, M. , Sabatini, A. , della Torre, A. , Di Deco, M. A. , & Petrarca, V. (2002). A polytene chromosome analysis of the *Anopheles gambiae* species complex. Science, 298, 1415–1418. 12364623 10.1126/science.1077769

[eva12517-bib-0015] Cornel, A. J. , & Collins, F. H. (1996). PCR of the ribosomal DNA intergenic spacer regions as a method for identifying mosquitoes in the *Anopheles gambiae* complex. Methods in Molecular Biology, 50, 321–332. 8751368 10.1385/0-89603-323-6:321

[eva12517-bib-0016] Costantini, C. , Ayala, D. , Guelbeogo, W. M. , Pombi, M. , Some, C. Y. , Bassole, I. H. , … Simard, F. (2009). Living at the edge: Biogeographic patterns of habitat segregation conform to speciation by niche expansion in *Anopheles gambiae* . BMC Ecology, 9(1), 16. 19460144 10.1186/1472-6785-9-16 PMC2702294

[eva12517-bib-0017] Coyne, J. A. , & Orr, H. A. (1989). Patterns of speciation in *Drosophila* . Evolution, 43(2), 362–381. 28568554 10.1111/j.1558-5646.1989.tb04233.x

[eva12517-bib-0018] Coyne, J. A. , & Orr, H. A. (1997). Patterns of speciation in *Drosophila* revisited. Evolution, 51(1), 295–303. 28568795 10.1111/j.1558-5646.1997.tb02412.x

[eva12517-bib-0019] Coyne, J. A. , & Orr, H. A. (1998). The evolutionary genetics of speciation. Philosophical Transactions of the Royal Society of London. Series B, Biological Sciences, 353(1366), 287–305. 9533126 10.1098/rstb.1998.0210 PMC1692208

[eva12517-bib-0503] Coyne, J. A. , & Orr, H. A. (2004). Speciation. Sunderland, MA: Sinauer Associates Inc., 545 pp.

[eva12517-bib-0020] Dabire, K. R. , Sawadodgo, S. , Diabate, A. , Toe, K. H. , Kengne, P. , Ouari, A. , … Gibson, G. (2013). Assortative mating in mixed swarms of the mosquito *Anopheles gambiae s.s*. M and S molecular forms, in Burkina Faso, West Africa. Medical and Veterinary Entomology, 27(3), 298–312. 23360106 10.1111/j.1365-2915.2012.01049.x

[eva12517-bib-0021] Dao, A. , Yaro, A. S. , Diallo, M. , Timbiné, S. , Huestis, D. L. , Kassogué, Y. , … Lehmann, T. (2014). Signatures of aestivation and migration in Sahelian malaria mosquito populations. Nature, 516(7531), 387–390. 25470038 10.1038/nature13987 PMC4306333

[eva12517-bib-0022] della Torre, A. , Fanello, C. , Akogbeto, M. , Dossou‐yovo, J. , Favia, G. , Petrarca, V. , & Coluzzi, M. (2001). Molecular evidence of incipient speciation within *Anopheles gambiae s.s*. in West Africa. Insect Molecular Biology, 10(1), 9–18. 11240632 10.1046/j.1365-2583.2001.00235.x

[eva12517-bib-0023] Diabaté, A. , Dabire, R. K. , Kim, E. H. , Dalton, R. , Millogo, N. , Baldet, T. , … Lehmann, T. (2005). Larval development of the molecular forms of *Anopheles gambiae* (Diptera: Culicidae) in different habitats: A transplantation experiment. Journal of Medical Entomology, 42(4), 548–553. 16119542 10.1093/jmedent/42.4.548

[eva12517-bib-0502] Diabaté, A. , Dabire, R. K. , Millogo, N. , Lehmann, T. (2007). Evaluating the effect of postmating isolation between molecular forms of Anopheles gambiae (Diptera: Culicidae). Journal of Medical Entomology, 44(1), 60–64. 17294921 10.1603/0022-2585(2007)44[60:eteopi]2.0.co;2

[eva12517-bib-0024] Diabaté, A. , Dao, A. , Yaro, A. S. , Adamou, A. , Gonzalez, R. , Manoukis, N. C. , … Lehmann, T. (2009). Spatial swarm segregation and reproductive isolation between the molecular forms of *Anopheles gambiae* . Proceedings. Biological Sciences/The Royal Society, 276(1676), 4215–4222. 10.1098/rspb.2009.1167 PMC2821344 19734189

[eva12517-bib-0025] Djogbénou, L. , Chandre, F. , Berthomieu, A. , Dabiré, R. , Koffi, A. , Alout, H. , & Weill, M. (2008). Evidence of introgression of the ace‐1(R) mutation and of the ace‐1 duplication in West African *Anopheles gambiae s.s* . PLoS ONE, 3(5), e2172. 18478097 10.1371/journal.pone.0002172 PMC2377098

[eva12517-bib-0026] Fanello, C. , Petrarca, V. , della Torre, A. , Santolamazza, F. , Dolo, G. , Coulibaly, M. , … Coluzzi, M. (2003). The pyrethroid knock‐down resistance gene in the *Anopheles gambiae* complex in Mali and further indication of incipient speciation within *An. gambiae s.s* . Insect Molecular Biology, 12(3), 241–245. 12752657 10.1046/j.1365-2583.2003.00407.x

[eva12517-bib-0027] Favia, G. , Lanfrancotti, A. , Spanos, L. , Sidén‐Kiamos, I. , & Louis, C. (2001). Molecular characterization of ribosomal DNA polymorphisms discriminating among chromosomal forms of *Anopheles gambiae s.s* . Insect Molecular Biology, 10(1), 19–23. 11240633 10.1046/j.1365-2583.2001.00236.x

[eva12517-bib-0028] Fontaine, M. C. , Pease, J. B. , Steele, A. , Waterhouse, R. M. , Neafsey, D. E. , Sharakhov, I. V. , … Besansky, N. J. (2015). Extensive introgression in a malaria vector species complex revealed by phylogenomics. Science, 347(6217), 1–14. 10.1126/science.1258524 PMC4380269 25431491

[eva12517-bib-0030] Funk, D. J. , Nosil, P. , & Etges, W. J. (2006). Ecological divergence exhibits consistently positive associations with reproductive isolation across disparate taxa. Proceedings of the National Academy of Sciences of the United States of America, 103(9), 3209–3213. 16492742 10.1073/pnas.0508653103 PMC1413886

[eva12517-bib-0031] Gimonneau, G. , Pombi, M. , Choisy, M. , Morand, S. , Dabiré, R. K. , & Simard, F. (2012). Larval habitat segregation between the molecular forms of the mosquito *Anopheles gambiae* in a rice field area of Burkina Faso, West Africa. Medical and Veterinary Entomology, 26(1), 9–17. 21501199 10.1111/j.1365-2915.2011.00957.x PMC3140611

[eva12517-bib-0032] Hahn, M. W. , White, B. J. , Muir, C. D. , & Besansky, N. J. (2012). No evidence for biased co‐transmission of speciation islands in *Anopheles gambiae* . Philosophical Transactions of the Royal Society of London. Series B, Biological Sciences, 367(1587), 374–384. 22201167 10.1098/rstb.2011.0188 PMC3233708

[eva12517-bib-0033] Higgins, J. P. , Thompson, S. G. , Deeks, J. J. , & Altman, D. G. (2003). Measuring inconsistency in meta‐analyses. BMJ (Clinical Research Ed.), 327(7414), 557–560. 10.1136/bmj.327.7414.557 PMC192859 12958120

[eva12517-bib-0034] Hui, W. , Gel, Y. , & Gastwirth, J. (2008). lawstat: An R package for law, public policy and biostatistics. Journal of Statistical Software, 28(3).

[eva12517-bib-0035] Kamali, M. , Xia, A. , Tu, Z. , & Sharakhov, I. V. (2012). A new chromosomal phylogeny supports the repeated origin of vectorial capacity in malaria mosquitoes of the *Anopheles gambiae* complex. PLoS Pathogens, 8(10), e1002960. 23055932 10.1371/journal.ppat.1002960 PMC3464210

[eva12517-bib-0036] Kamdem, C. , Tene Fossog, B. , Simard, F. , Etouna, J. , Ndo, C. , Kengne, P. , … Costantini, C. (2012). Anthropogenic habitat disturbance and ecological divergence between incipient species of the malaria mosquito *Anopheles gambiae* . PLoS ONE, 7(6), e39453. 22745756 10.1371/journal.pone.0039453 PMC3382172

[eva12517-bib-0037] Kirkpatrick, M. (2001). Reinforcement during ecological speciation. Proceedings. Biological Sciences/The Royal Society, 268(1473), 1259–1263. 10.1098/rspb.2000.1427 PMC1088735 11410152

[eva12517-bib-0038] Kitau, J. , Oxborough, R. M. , Tungu, P. K. , Matowo, J. , Malima, R. C. , Magesa, S. M. , … Rowland, M. W. (2012). Species shifts in the *Anopheles gambiae* complex: Do LLINs successfully control *Anopheles arabiensis*? PLoS ONE, 7(3), 1–7. 10.1371/journal.pone.0031481 PMC3306310 22438864

[eva12517-bib-0039] Knols, B. G. J. , Bossin, H. C. , Mukabana, W. R. , & Robinson, A. S. (2007). Transgenic mosquitoes and the fight against malaria: Managing technology push in a turbulent GMO world. American Journal of Tropical Medicine and Hygiene, 77(Suppl. 6), 232–242. 18165498

[eva12517-bib-0040] Lawniczak, M. K. , Emrich, S. J. , Holloway, A. K. , Regier, A. P. , Olson, M. , White, B. , … Besansky, N. J. (2010). Widespread divergence between incipient *Anopheles gambiae* species revealed by whole genome sequences. Science, 330(6003), 512–514. 20966253 10.1126/science.1195755 PMC3674514

[eva12517-bib-0041] Lee, Y. , Cornel, A. J. , Meneses, C. R. , Fofana, A. , Andrianarivo, A. G. , McAbee, R. D. , … Lanzaro, G. C. (2009). Ecological and genetic relationships of the Forest‐M form among chromosomal and molecular forms of the malaria vector *Anopheles gambiae* sensu stricto. Malaria Journal, 8, 75. 19383163 10.1186/1475-2875-8-75 PMC2680901

[eva12517-bib-0042] Lee, Y. , Marsden, C. D. , Norris, L. C. , Collier, T. C. , Main, B. J. , Fofana, A. , … Lanzaro, G. C. (2013). Spatiotemporal dynamics of gene flow and hybrid fitness between the M and S forms of the malaria mosquito, *Anopheles gambiae* . Proceedings of the National Academy of Sciences of the United States of America, 110(49), 19854–19859. 24248386 10.1073/pnas.1316851110 PMC3856788

[eva12517-bib-0043] Lehmann, T. , & Diabate, A. (2008). The molecular forms of *Anopheles gambiae*: A phenotypic perspective. Infection, Genetics and Evolution, 8(5), 737–746. 10.1016/j.meegid.2008.06.003 PMC2731232 18640289

[eva12517-bib-0044] Mallet, J. (2005). Hybridization as an invasion of the genome. Trends in Ecology & Evolution, 20(5), 229–237. 16701374 10.1016/j.tree.2005.02.010

[eva12517-bib-0045] Mallet, J. (2006). What does *Drosophila* genetics tell us about speciation? Trends in Ecology & Evolution, 21(7), 386–393. 16765478 10.1016/j.tree.2006.05.004

[eva12517-bib-0046] Mancini, E. , Spinaci, M. I. , Gordicho, V. , Caputo, B. , Pombi, M. , Vicente, J. L. , … della Torre, A. (2015). Adaptive potential of hybridization among malaria vectors: Introgression at the immune locus TEP1 between *Anopheles coluzzii* and *A. gambiae* in “Far‐West” Africa. PLoS ONE, 10(6), 1–13. 10.1371/journal.pone.0127804 PMC4457524 26047479

[eva12517-bib-0047] Marsden, C. D. , Lee, Y. , Nieman, C. C. , Sanford, M. R. , Dinis, J. , Martins, C. , … Lanzaro, G. C. (2011). Asymmetric introgression between the M and S forms of the malaria vector, *Anopheles gambiae*, maintains divergence despite extensive hybridization. Molecular Ecology, 20(23), 4983–4994. 22059383 10.1111/j.1365-294X.2011.05339.x PMC3222736

[eva12517-bib-0049] McBride, C. S. , & Singer, M. C. (2010). Field studies reveal strong postmating isolation between ecologically divergent butterfly populations. PLoS Biology, 8(10), e1000529. 21048982 10.1371/journal.pbio.1000529 PMC2964332

[eva12517-bib-0050] Neafsey, D. E. , Lawniczak, M. K. , Park, D. J. , Redmond, S. N. , Coulibaly, M. B. , Traoré, S. F. , … Muskavitch, M. A. (2010). SNP genotyping defines complex gene‐flow boundaries among African malaria vector mosquitoes. Science, 330(6003), 514–517. 20966254 10.1126/science.1193036 PMC4811326

[eva12517-bib-0051] Niang, A. , Epopa, P. S. , Sawadogo, S. P. , Maïga, H. , Konaté, L. , Faye, O. , … Diabaté, A. (2015). Does extreme asymmetric dominance promote hybridization between Anopheles coluzzii and *Anopheles gambiae* s.s. in seasonal malaria mosquito communities of West Africa?. Parasites & Vectors, 8(1), 586. 26559354 10.1186/s13071-015-1190-x PMC4642620

[eva12517-bib-0052] Noor, M. A. (1997). How often does sympatry affect sexual isolation in *Drosophila*? The American Naturalist, 149(6), 1156–1163. 10.1086/286044 18811269

[eva12517-bib-0053] Norris, L. C. , Main, B. J. , Lee, Y. , Collier, T. C. , Fofana, A. , Cornel, A. J. , & Lanzaro, G. C. (2015). Adaptive introgression in an African malaria mosquito coincident with the increased usage of insecticide‐treated bed nets. Proceedings of the National Academy of Sciences of the United States of America, 112(3), 815–820. 25561525 10.1073/pnas.1418892112 PMC4311837

[eva12517-bib-0054] Nosil, P. , & Crespi, B. J. (2006). Experimental evidence that predation promotes divergence in adaptive radiation. Proceedings of the National Academy of Sciences of the United States of America, 103(24), 9090–9095. 16754870 10.1073/pnas.0601575103 PMC1482571

[eva12517-bib-0055] Oliveira, E. , Salgueiro, P. , Palsson, K. , Vicente, J. L. , Arez, A. P. , Jaenson, T. G. , … Pinto, J. (2008). High levels of hybridization between molecular forms of *Anopheles gambiae* from Guinea Bissau. Journal of Medical Entomology, 45(6), 1057–1063. 19058629 10.1603/0022-2585(2008)45[1057:hlohbm]2.0.co;2

[eva12517-bib-0056] Orr, H. A. , & Coyne, J. A. (1989). The genetics of postzygotic isolation in the *Drosophila virilis* group. Genetics, 121(3), 527–537. 2714637 10.1093/genetics/121.3.527 PMC1203638

[eva12517-bib-0057] Pennetier, C. , Warren, B. , Dabiré, K. R. , Russell, I. J. , & Gibson, G. (2010). “Singing on the wing” as a mechanism for species recognition in the malarial mosquito *Anopheles gambiae* . Current Biology, 20(2), 131–136. 20045329 10.1016/j.cub.2009.11.040

[eva12517-bib-0058] Pérez‐Figueroa, A. , Cruz, F. , Carvajal‐Rodríguez, A. , Rolán‐Alvarez, E. , & Caballero, A. (2005). The evolutionary forces maintaining a wild polymorphism of *Littorina saxatilis*: Model selection by computer simulations. Journal of Evolutionary Biology, 18(1), 191–202. 15669976 10.1111/j.1420-9101.2004.00773.x

[eva12517-bib-0059] Pianka, E. R. (1974). Niche overlap and diffuse competition. Proceedings of the National Academy of Sciences of the United States of America, 71(5), 2141–2145. 4525324 10.1073/pnas.71.5.2141 PMC388403

[eva12517-bib-0060] Pinto, J. , Egyir‐Yawson, A. , Vicente, J. L. , Gomes, B. , Santolamazza, F. , Moreno, M. , … della Torre, A. (2013). Geographic population structure of the African malaria vector *Anopheles gambiae* suggests a role for the forest‐savannah biome transition as a barrier to gene flow. Evolutionary Applications, 6(6), 910–924. 24062800 10.1111/eva.12075 PMC3779092

[eva12517-bib-0061] Price, C. S. , Kim, C. H. , Gronlund, C. J. , & Coyne, J. A. (2001). Cryptic reproductive isolation in the *Drosophila simulans* species complex. Evolution, 55(1), 81–92. 11263748 10.1111/j.0014-3820.2001.tb01274.x

[eva12517-bib-0062] R Core Team (2013). R: A language and environment for statistical computing. Vienna, Austria: R Foundation for Statistical Computing.

[eva12517-bib-0063] Racimo, F. , Sankararaman, S. , Nielsen, R. , & Huerta‐Sánchez, E. (2015). Evidence for archaic adaptive introgression in humans. Nature Reviews Genetics, 16(6), 359–371. 10.1038/nrg3936 PMC4478293 25963373

[eva12517-bib-0064] Reidenbach, K. R. , Neafsey, D. E. , Costantini, C. , Sagnon, N. , Simard, F. , Ragland, G. J. , … Besansky, N. J. (2012). Patterns of genomic differentiation between ecologically differentiated M and S forms of *Anopheles gambiae* in West and Central Africa. Genome Biology and Evolution, 4(12), 1202–1212. 23132896 10.1093/gbe/evs095 PMC3542583

[eva12517-bib-0065] Rice, W. R. , & Hostert, E. E. (1993). Laboratory experiments on speciation: What have we learned in forty years? Evolution, 47(6), 1637–1653. 28568007 10.1111/j.1558-5646.1993.tb01257.x

[eva12517-bib-0066] Ridley, C. E. , & Alexander, L. C. (2016). Applying gene flow science to environmental policy needs: A boundary work perspective. Evolutionary Applications, 9(7), 924–936. 27468309 10.1111/eva.12393 PMC4947153

[eva12517-bib-0067] Riehle, M. M. , Guelbeogo, W. M. , Gneme, A. , Eiglmeier, K. , Holm, I. , Bischoff, E. , … Vernick, K. D. (2011). A cryptic subgroup of *Anopheles gambiae* is highly susceptible to human malaria parasites. Science, 331(6017), 596–598. 21292978 10.1126/science.1196759 PMC3065189

[eva12517-bib-0068] Rundle, H. D. , & Nosil, P. (2005). Ecological speciation. Ecology Letters, 8(3), 336–352.

[eva12517-bib-0069] Russell, T. L. , Govella, N. J. , Azizi, S. , Drakeley, C. J. , Kachur, S. P. , & Killeen, G. F. (2011). Increased proportions of outdoor feeding among residual malaria vector populations following increased use of insecticide‐treated nets in rural Tanzania. Malaria Journal, 10(1), 80. 21477321 10.1186/1475-2875-10-80 PMC3084176

[eva12517-bib-0070] Santolamazza, F. , Caputo, B. , Calzetta, M. , Vicente, J. L. , Mancini, E. , Petrarca, V. , … della Torre, A. (2011). Comparative analyses reveal discrepancies among results of commonly used methods for *Anopheles gambiae* molecular form identification. Malaria Journal, 10(1), 215. 21810255 10.1186/1475-2875-10-215 PMC3170251

[eva12517-bib-0071] Santolamazza, F. , Mancini, E. , Simard, F. , Qi, Y. , Tu, Z. , … della Torre, A. (2008). Insertion polymorphisms of SINE200 retrotransposons within speciation islands of *Anopheles gambiae* molecular forms. Malaria Journal, 7(1), 163. 18724871 10.1186/1475-2875-7-163 PMC2546427

[eva12517-bib-0072] Sawadogo, S. P. , Costantini, C. , Pennetier, C. , Diabaté, A. , Gibson, G. , & Dabiré, R. K. (2013). Differences in timing of mating swarms in sympatric populations of Anopheles coluzzii and *Anopheles gambiae* s.s. (formerly *An. gambiae* M and S molecular forms) in Burkina Faso, West Africa. Parasites & Vectors, 6, 275. 24330578 10.1186/1756-3305-6-275 PMC3851435

[eva12517-bib-0073] Sawadogo, S. P. , Namountougou, M. , Toé, K. H. , Rouamba, J. , Maïga, H. , Ouédraogo, K. R. , … Dabiré, K. R. (2014). Swarming behaviour in natural populations of *Anopheles gambiae* and *An. coluzzii*: Review of 4 years survey in rural areas of sympatry, Burkina Faso (West Africa). Acta Tropica, 130(1), 24–34. 10.1016/j.actatropica.2013.12.011 24370676

[eva12517-bib-0074] Schluter, D. , & Conte, G. L. (2009). Genetics and ecological speciation. Proceedings of the National Academy of Sciences of the United States of America, 106(Suppl.), 9955–9962. 19528639 10.1073/pnas.0901264106 PMC2702799

[eva12517-bib-0075] Scott, J. A. , Brogdon, W. G. , & Collins, F. H. (1993). Identification of single specimens of the *Anopheles gambiae* complex by the polymerase chain reaction. American Journal of Tropical Medicine and Hygiene, 49(4), 520–529. 8214283 10.4269/ajtmh.1993.49.520

[eva12517-bib-0076] Scott, T. W. , Takken, W. , Knols, B. G. J. , & Boete, C. (2002). The ecology of genetically modified mosquitoes. Science, 298(5591), 117–119. 12364785 10.1126/science.298.5591.117

[eva12517-bib-0077] Seehausen, O. (2004). Hybridization and adaptive radiation. Trends in Ecology & Evolution, 19(4), 198–207. 16701254 10.1016/j.tree.2004.01.003

[eva12517-bib-0078] Seehausen, O. (2013). Conditions when hybridization might predispose populations for adaptive radiation. Journal of Evolutionary Biology, 26(2), 279–281. 23324007 10.1111/jeb.12026

[eva12517-bib-0079] Sharakhov, I. V. , White, B. J. , Sharakhova, M. V. , Kayondo, J. , Lobo, N. F. , Santolamazza, F. , … Besansky, N. J. (2006). Breakpoint structure reveals the unique origin of an interspecific chromosomal inversion (2La) in the *Anopheles gambiae* complex. Proceedings of the National Academy of Sciences of the United States of America, 103(16), 6258–6262. 16606844 10.1073/pnas.0509683103 PMC1458865

[eva12517-bib-0080] Simard, F. , Ayala, D. , Kamdem, C. , Pombi, M. , Etouna, J. , Ose, K. , … Costantini, C. (2009). Ecological niche partitioning between *Anopheles gambiae* molecular forms in Cameroon: The ecological side of speciation. BMC Ecology, 9(1), 17. 19460146 10.1186/1472-6785-9-17 PMC2698860

[eva12517-bib-0081] Simões, P. M. , Gibson, G. , & Russell, I. J. (2017). Pre‐copula acoustic behaviour of males in the malarial mosquitoes *Anopheles coluzzii* and *Anopheles gambiae s.s*. does not contribute to reproductive isolation. The Journal of Experimental Biology, 220(Pt 3), 379–385. 28148817 10.1242/jeb.149757

[eva12517-bib-0082] Slotman, M. A. , della Torre, A. , Calzetta, M. , & Powell, J. R. (2005). Differential introgression of chromsomal regions between *Anopheles gambiae* and *An. arabiensis* . The American Journal of Tropical Medicine and Hygiene, 73(2), 326–335. 16103599

[eva12517-bib-0083] Slotman, M. A. , della Torre, A. , & Powell, J. R. (2004). The genetics of inviability and male sterility in hybrids between *Anopheles gambiae* and *An. arabiensis* . Genetics, 287(1), 275–287. 10.1534/genetics.167.1.275 PMC1470845 15166154

[eva12517-bib-0084] Slotman, M. A. , Reimer, L. J. , Thiemann, T. , Dolo, G. , Fondjo, E. , & Lanzaro, G. C. (2006). Reduced recombination rate and genetic differentiation between the M and S forms of *Anopheles gambiae s.s* . Genetics, 174(4), 2081–2093. 17057242 10.1534/genetics.106.059949 PMC1698612

[eva12517-bib-0085] Sobel, J. M. , & Chen, G. F. (2014). Unification of methods for estimating the strength of reproductive isolation. Evolution, 68(5), 1511–1522. 24450287 10.1111/evo.12362

[eva12517-bib-0086] Sobel, J. M. , Chen, G. F. , Watt, L. R. , & Schemske, D. W. (2010). The biology of speciation. Evolution, 64(2), 295–315. 19891628 10.1111/j.1558-5646.2009.00877.x

[eva12517-bib-0087] Sobel, J. M. , & Streisfeld, M. A. (2015). Strong premating reproductive isolation drives incipient speciation in *Mimulus aurantiacus* . Evolution, 69(2), 447–461. 25545789 10.1111/evo.12589

[eva12517-bib-0088] Stankowski, S. (2013). Ecological speciation in an island snail: Evidence for the parallel evolution of a novel ecotype and maintenance by ecologically dependent postzygotic isolation. Molecular Ecology, 22(10), 2726–2741. 23506623 10.1111/mec.12287

[eva12517-bib-0501] Tene Fossog, B. , Ayala, D. , Acevedo, P. , Kengne, P. , Ngomo Abeso Mebuy, I. , Makanga, B. , … Costantini, C. (2014). Habitat segregation and ecological character displacement in cryptic African malaria mosquitoes. Evolutionary Applications, 8(4), 326–345. 10.1111/eva.12242 PMC4408144 25926878

[eva12517-bib-0089] The Anopheles gambiae 1000 Genomes Consortium (2016). Natural diversity of the malaria vector Anopheles gambiae. Retrieved from https://doi.org/10.1101/096289

[eva12517-bib-0090] Touré, Y. T. , Petrarca, V. , Traoré, S. F. , Coulibaly, A. , Maiga, H. M. , Sankaré, O. , … Coluzzi, M. (1998). The distribution and inversion polymorphism of chromosomally recognized taxa of the *Anopheles gambiae* complex in Mali, West Africa. Parassitologia, 40(4), 477–511. 10645562

[eva12517-bib-0091] Tripet, F. , Touré, Y. T. , Taylor, C. E. , Norris, D. E. , Dolo, G. , & Lanzaro, G. C. (2001). DNA analysis of transferred sperm reveals significant levels of gene flow between molecular forms of *Anopheles gambiae* . Molecular Ecology, 10(7), 1725–1732. 11472539 10.1046/j.0962-1083.2001.01301.x

[eva12517-bib-0092] Turelli, M. , Lipkowitz, J. R. , & Brandvain, Y. (2014). On the Coyne and Orr‐igin of species: Effects of intrinsic postzygotic isolation, ecological differentiation, X chromosome size, and sympatry on *Drosophila* speciation. Evolution, 68(4), 1176–1187. 24325145 10.1111/evo.12330 PMC4142334

[eva12517-bib-0093] Ulrich, W. , & Gotelli, N. J. (2010). Null model analysis of species associations using abundance data. Ecology, 91(11), 3384–3397. 21141199 10.1890/09-2157.1

[eva12517-bib-0094] Vicente, J. L. , Clarkson, C. L. , Caputo, B. , Gomes, B. , Pombi, M. , Sousa, C. A. , … Pinto, J. (2017). Is massive introgression driving species collapse or radiation at the range limit of *Anopheles gambiae*? Scientific Reports, 7, 46451. 28417969 10.1038/srep46451 PMC5394460

[eva12517-bib-0095] Viechtbauer, W. (2010). Conducting meta‐analyses in R with the metafor Package. Journal of Statistical Software, 36(3), 1–48.

[eva12517-bib-0096] Walsh, P. S. , Metzger, D. A. , & Higuchi, R. (1991). Chelex 100 as a medium for simple extraction of DNA for PCR‐based typing from forensic material. BioTechniques, 10(4), 506–513. 1867860

[eva12517-bib-0097] Weetman, D. , Wilding, C. S. , Steen, K. , Pinto, J. , & Donnelly, M. J. (2012). Gene flow‐dependent genomic divergence between *Anopheles gambiae* M and S forms. Molecular Biology and Evolution, 29(1), 279–291. 21836185 10.1093/molbev/msr199 PMC3259608

[eva12517-bib-0098] White, G. B. (1971). Chromosomal evidence for natural interspecific hybridization by mosquitoes of the *Anopheles gambiae* complex. Nature, 231(5299), 184–185. 4930676 10.1038/231184a0

[eva12517-bib-0099] White, G. B. (1974). *Anopheles gambiae* complex and disease transmission in Africa. Transactions of the Royal Society of Tropical Medicine and Hygiene, 68(4), 278–301. 4420769 10.1016/0035-9203(74)90035-2

[eva12517-bib-0100] White, B. J. , Lawniczak, M. K. N. , Cheng, C. , Coulibaly, M. B. , Wilson, M. D. , Sagnon, N. , … Besansky, N. J. (2011). Adaptive divergence between incipient species of *Anopheles gambiae* increases resistance to *Plasmodium* . Proceedings of the National Academy of Sciences of the United States of America, 108(1), 244–249. 21173248 10.1073/pnas.1013648108 PMC3017163

[eva12517-bib-0101] WHO (2016). World Malaria report. World Health Organization.

[eva12517-bib-0102] Wood, S. N. (2006). Generalized additive models: An introduction with R. Boca Raton, FL: Chapman & Hall/CRC.

[eva12517-bib-0103] Zhang, J. T. (2004). Quantitative ecology. Beijing, China: Science Press.

