# Supplementary material for: Dissecting functional components of reproductive isolation among closely related sympatric species of the Anopheles gambiae complex
Source: Evol Appl. 2017 Oct 5;10(10):1102–20. doi: 10.1111/eva.12517 (PMC5680640; doi:10.1111/eva.12517)
Supplement: Supplementary file 1 [file EVA-10-1102-s001.docx]

### **Appendix S1**

### Possible confounding taxa in Burkina Faso

A fourth cryptic taxon belonging to the *An. gambiae* complex, known as *An. gambiae* GOUNDRY, has been identified from larval samples of *An. gambiae* (Giles) occurring exclusively—as far as is known—in the arid savannah of Burkina Faso (Riehle *et al.* 2011). This taxon is polymorphic for the rDNA IGS diagnostic markers of *An. coluzzii* and *An. gambiae* *s.s.*, and was distinguished based on Bayesian multilocus assignment of microsatellite alleles located on chromosome 3. In population samples where this taxon is sufficiently abundant, therefore, many individuals may misleadingly appear as *An. coluzzii* × *gambiae* *s.s.* ‘hybrids’ (in Hardy-Weinberg equilibrium) when only the IGS diagnostic test is applied. *Anopheles gambiae* GOUNDRY—hereafter also GOUNDRY as a shorthand—is suspected to rest exclusively outdoors (Riehle *et al.* 2011), so it is likely that our samples did not include consequential amounts—if any—of GOUNDRY mosquitoes. In fact, the data set we gathered from Burkina Faso is constituted solely of samples of indoor-resting *An. gambiae* *s.l.* However, because the presence of the GOUNDRY taxon can confound our hybridization analysis, here we present also data of larval and adult outdoor-resting *An. gambiae* *s.l.* collected in or near the Goundry village (from which this taxon has been originally identified), providing clues about the abundance of the GOUNDRY taxon in natural field populations.

Table S7 reports the results of molecular identifications of 4,270 *An. gambiae* (Giles) larvae sampled from more than 350 different larval habitats in Burkina Faso during the period 1996-2009. Most of these samples come from the same locality where GOUNDRY has been recorded. However, despite the potential occurrence of this taxon in our samples, the overall frequency of larvae returning the electropherogram pattern of *An. coluzzii* × *gambiae* *s.s.* hybrids by the IGS molecular diagnostic tests of Favia *et al.* (2001) or Fanello *et al.* (2003) was only 0.18% (doubled with respect to the observed average value, due to non-detectability of hybrid male larvae). Similarly, no hybrid electromorphs could be detected in samples collected during the 1998 rainy season in the village of Goundry from pit-shelters (*n*=239) or landing on humans out-of-doors (*n*=263). Moreover, Pombi *et al.* (2014, 2015) conducted extensive parallel indoor/outdoor surveys of members of the *An. gambiae* complex in Goden and Koubri (10 Km and 40 Km from Goundry, respectively, as the bee flies) throughout both rainy and dry seasons in 2011-2012. Only 0.17% of 10,367 *An. gambiae* *s.l.* specimens returned the *An. coluzzii* × *gambiae* *s.s.* hybrid pattern of the IGS diagnostic. Taken together, these observations suggest that the GOUNDRY taxon is extremely rare and/or sparsely distributed in space and/or time to be often below the threshold of detection in population samples of *An. gambiae* *s.l.*, perhaps reflecting the availability of quite specific, but presently unknown, environmental conditions. Alternatively, collecting devices and methods used routinely to sample members of the *An. gambiae* complex may be extremely inefficient in detecting this taxon. Either way, the occurrence of GOUNDRY in the study area is unlikely to have unduly affected our hybridization analysis.

**Table S7. Frequency of hybrids between *An. coluzzii* and *An. An. gambiae s.s.* in larval samples from Burkina Faso.**

| **Locality / Year** | **No. Hybrids (%)** | | **No. Specimens** | **Source** |
| --- | --- | --- | --- | --- |
| Goundry 1996 | 0 | (0.00%) | 169 | Sagnon 1999, PhD thesis, Univ. Rome “La Sapienza” |
| Goundry 1998 | 0 | (0.00%) | 165 | Unpublished data |
| Goundry 2000 | 2 | (0.25%) | 796 | Unpublished data |
| Goundry 2003 | 0 | (0.00%) | 2,522 | Pombi 2004, PhD thesis, Univ. Rome “La Sapienza” |
| Bama 2009 | 2 | (0.32%) | 618 | Gimonneau *et al.* (2012) |
| Total | 4 | (0.09%) | 4,270 |  |

**References**

Fanello, C., V. Petrarca, A. della Torre, F. Santolamazza, G. Dolo, M. Coulibaly, A. Alloueche, C. F. Curtis, Y. T. Touré, and M. Coluzzi. 2003. The pyrethroid knock-down resistance gene in the *Anopheles gambiae* complex in Mali and further indication of incipient speciation within *An. gambiae s.s. Insect Mol. Biol.* 12:241–5.

Favia, G., A. Lanfrancotti, L. Spanos, I. Sidén-Kiamos, and C. Louis. 2001. Molecular characterization of ribosomal DNA polymorphisms discriminating among chromosomal forms of *Anopheles gambiae s.s. Insect Mol. Biol.* 10:19–23.

Pombi, M., W. M. Guelbeogo, M. Calzetta, N. Sagnon, V. Petrarca, V. La Gioia, and A. della Torre. 2015. Evaluation of a protocol for remote identification of mosquito vector species reveals BG-Sentinel trap as an efficient tool for *Anopheles gambiae* outdoor collection in Burkina Faso. *Malar. J.* 14:161.

Pombi, M., W. M. Guelbeogo, K. Kreppel, M. Calzetta, A. Traoré, A. Sanou, H. Ranson, H. M. Ferguson, N. Sagnon, and A. della Torre. 2014. The Sticky Resting Box, a new tool for studying resting behaviour of Afrotropical malaria vectors. *Parasit. Vectors* 7:247.

Riehle, M. M., W. M. Guelbeogo, A. Gneme, K. Eiglmeier, I. Holm, E. Bischoff, T. Garnier, G. M. Snyder, X. Li, K. Markianos, N. Sagnon, and K. D. Vernick. 2011. A cryptic subgroup of *Anopheles gambiae* is highly susceptible to human malaria parasites. *Science* 331:596–8.
