# Supplementary material for: Dissecting functional components of reproductive isolation among closely related sympatric species of the Anopheles gambiae complex
Source: Evol Appl. 2017 Oct 5;10(10):1102–20. doi: 10.1111/eva.12517 (PMC5680640; doi:10.1111/eva.12517)
Supplement: Supplementary file 2 [file EVA-10-1102-s002.docx]

**Appendix S2**

List of published articles retained from a systematic review of field studies indexed in the publication repository PubMed (<http://www.ncbi.nlm.nih.gov/pubmed>). See main text for details.

1. Antonio-Nkondjio C, Defo-Talom B, Tagne-Fotso R, Tene-Fossog B, Ndo C, et al. (2012) High mosquito burden and malaria transmission in a district of the city of Douala, Cameroon. BMC Infect Dis 12: 275.

2. Antonio-Nkondjio C, Demanou M, Etang J, Bouchite B (2013) Impact of cyfluthrin (Solfac EW050) impregnated bed nets on malaria transmission in the city of Mbandjock : lessons for the nationwide distribution of long-lasting insecticidal nets (LLINs) in Cameroon. Parasit Vectors 6: 10.

3. Appawu M, Baffoe-Wilmot A, Afari E (1994) Species composition and inversion polymorphism of the Anopheles gambiae complex in some sites of Ghana, West Africa. Acta Trop 56.

4. Appawu M, Owusu-Agyei S, Dadzie S, Asoala V, Anto F, et al. (2004) Malaria transmission dynamics at a site in northern Ghana proposed for testing malaria vaccines. Trop Med Int Health 9: 164–170.

5. Awolola TS, Oyewole IO, Amajoh CN, Idowu ET, Ajayi MB, et al. (2005) Distribution of the molecular forms of Anopheles gambiae and pyrethroid knock down resistance gene in Nigeria. Acta Trop 95: 204–209.

6. Awolola TS, Oduola AO, Obansa JB, Chukwurar NJ, Unyimadu JP (2007) Anopheles gambiae s.s. breeding in polluted water bodies in urban Lagos, southwestern Nigeria. J Vector Borne Dis 44: 241–244.

7. Besansky NJ, Lehmann T, Fahey GT, Fontenille D, Braack LE, et al. (1997) Patterns of mitochondrial variation within and between African malaria vectors, Anopheles gambiae and An. arabiensis, suggest extensive gene flow. Genetics 147: 1817–1828.

8. Bøgh C, Clarke SE, Jawara M, Thomas CJ, Lindsay SW (2003) Localized breeding of the Anopheles gambiae complex (Diptera: Culicidae) along the River Gambia, West Africa. Bull Entomol Res 93: 279–287.

9. Bryan JH, Petrarca V, Di Deco MA, Coluzzi M (1987) Adult behaviour of members of the Anopheles gambiae complex in the Gambia with special reference to An. melas and its chromosomal variants. Parassitologia 29: 221–249.

10. Bryan J (1979) Observations on the member species of the Anopheles gambiae complex in The Gambia, West Africa. Trans R Soc Trop Med Hyg 73: 463–466.

11. Calzetta M, Santolamazza F, Carrara GC, Cani PJ, Fortes F, et al. (2008) Distribution and chromosomal characterization of the Anopheles gambiae complex in Angola. Am J Trop Med Hyg 78: 169–175.

12. Caputo B, Dani FR, Horne GL, Sagnon N, Diabate A, et al. (2007) Comparative analysis of epicuticular lipid profiles of sympatric and allopatric field populations of Anopheles gambiae s.s. molecular forms and An. arabiensis from Burkina Faso (West Africa). Insect Biochem Mol Biol 37: 389–398.

13. Caputo B, Nwakanma D, Jawara M, Adiamoh M, Dia I, et al. (2008) Anopheles gambiae complex along The Gambia river, with particular reference to the molecular forms of An. gambiae s.s. Malar J 7: 182.

14. Chanda E, Hemingway J, Kleinschmidt I, Rehman AM, Ramdeen V, et al. (2011) Insecticide resistance and the future of malaria control in Zambia. PLoS One 6: e24336.

15. Charlwood JD, Tomás E V, Egyir-Yawson a, Kampango a a, Pitts RJ (2012) Feeding frequency and survival of Anopheles gambiae in a rice-growing area in Ghana. Med Vet Entomol 26: 263–270.

16. Choi KS, Townson H (2012) Evidence for X-linked introgression between molecular forms of Anopheles gambiae from Angola. Med Vet Entomol 26: 218–227.

17. Choi KS, Spillings BL, Coetzee M, Hunt RH, Koekemoer LL (2010) A comparison of DNA sequencing and the hydrolysis probe analysis (TaqMan assay) for knockdown resistance (kdr) mutations in Anopheles gambiae from the Republic of the Congo. Malar J 9: 278.

18. Coluzzi M, Sabatini A, Petrarca V (1975) Field research project in epidemiology and control of malaria in African savanna, Kano, (Nigeria). World Heal Organ Tech Notes 24: 16–25.

19. Coosemans M, Petrarca V, Barutwanayo M, Coluzzi M (1989) Species of the Anopheles gambiae complex and chromosomal polymorphism in a rice-growing area of the Rusizi Valley (Republic of Burundi). Parassitologia 31: 113–122.

20. Costantini C, Diallo M (2001) Preliminary lack of evidence for simian odour preferences of savanna populations of Anopheles gambiae and other malaria vectors. Parassitologia 43: 179–182.

21. Costantini C, Ayala D, Guelbeogo WM, Pombi M, Some CY, et al. (2009) Living at the edge: biogeographic patterns of habitat segregation conform to speciation by niche expansion in Anopheles gambiae. BMC Ecol 9: 16.

22. Cuamba N, Choi KS, Townson H (2006) Malaria vectors in Angola: distribution of species and molecular forms of the Anopheles gambiae complex, their pyrethroid insecticide knockdown resistance (kdr) status and Plasmodium falciparum sporozoite rates. Malar J 5: 2.

23. Dabiré KR, Diabaté a, Namontougou M, Djogbenou L, Kengne P, et al. (2009) Distribution of insensitive acetylcholinesterase (ace-1R) in Anopheles gambiae s.l. populations from Burkina Faso (West Africa). Trop Med Int Health 14: 396–403.

24. della Torre A, Fanello C, Akogbeto M, Dossou-yovo J, Favia G, et al. (2001) Molecular evidence of incipient speciation within Anopheles gambiae s.s. in West Africa. Insect Mol Biol 10: 9–18.

25. della Torre A, Tu Z, Petrarca V (2005) On the distribution and genetic differentiation of Anopheles gambiae s.s. molecular forms. Insect Biochem Mol Biol 35: 755–769. Field studies cited in Table 1:

- Adasi, Hemingway J, della Torre, unpublished
- Akogbeto, della Torre, unpublished
- Costantini, Sagnon, della Torre, unpublished
- Dia & della Torre, unpublished
- Medjibe, Caccone, Powell, della Torre, unpublished
- Takken, della Torre, Petrarca, unpublished

26. Dery DB, Brown C, Asante KP, Adams M, Dosoo D, et al. (2010) Patterns and seasonality of malaria transmission in the forest-savannah transitional zones of Ghana. Malar J 9: 314.

27. Dia I, Diop T, Rakotoarivony I, Kengne P, Fontenille D (2003) Bionomics of Anopheles gambiae Giles, An. arabiensis Patton, An. funestus Giles and An. nili (Theobald) (Diptera: Culicidae) and transmission of Plasmodium falciparum in a Sudano-Guinean zone (Ngari, Senegal). J Med Entomol 40: 279–283.

28. Diabaté A, Baldet T, Chandre F, Guiguemdé RT, Brengues C, et al. (2002) First report of the kdr mutation in Anopheles gambiae M form from Burkina Faso, west Africa. Parassitologia 44: 157–158.

29. Diabate A, Baldet T, Chandre F, Dabire RK, Kengne P, et al. (2003) Mutation, a genetic marker to assess events of introgression between the molecular M and S forms of Anopheles gambiae (Diptera: Culicidae) in the tropical savannah. J Med Entomol 40: 195–198.

30. Diatta M, Spiegel A, Lochouarn L, Fontenille D (1998) Similar feeding preferences of Anopheles gambiae and A. arabiensis in Senegal. Trans R Soc Trop Med Hyg: 270–272.

31. Duchemin JB, Leong Pock Tsy JM, Rabarison P, Roux J, Coluzzi M, et al. (2001) Zoophily of Anopheles arabiensis and An. gambiae in Madagascar demonstrated by odour-baited entry traps. Med Vet Entomol 15: 50–57.

32. Ebenezer a, Okiwelu SN, Agi PI, Noutcha M a E, Awolola TS, et al. (2012) Species composition of the Anopheles gambiae complex across eco-vegetational zones in Bayelsa State, Niger Delta region, Nigeria. J Vector Borne Dis 49: 164–167.

33. Edillo FE, Touré YT, Lanzaro GC, Dolo G, Taylor CE (2002) Spatial and habitat distribution of Anopheles gambiae and Anopheles arabiensis (Diptera: Culicidae) in Banambani village, Mali. J Med Entomol 39: 70–77.

34. Esnault C, Boulesteix M, Duchemin JB, Koffi A a, Chandre F, et al. (2008) High genetic differentiation between the M and S molecular forms of Anopheles gambiae in Africa. PLoS One 3: e1968.

35. Fanello C, Petrarca V, della Torre a, Santolamazza F, Dolo G, et al. (2003) The pyrethroid knock-down resistance gene in the Anopheles gambiae complex in Mali and further indication of incipient speciation within An. gambiae s.s. Insect Mol Biol 12: 241–245.

36. Favia G, Torre A Della (1997) Molecular identification of sympatric chromosomal forms of Anopheles gambiae and further evidence of their reproductive isolation. Insect Mol Biol 6: 377–383.

37. Fillinger U, Sombroek H, Majambere S, van Loon E, Takken W, et al. (2009) Identifying the most productive breeding sites for malaria mosquitoes in The Gambia. Malar J 8: 62.

38. Fontenille D, Lochouarn L, Diagne N, Sokhna C, Lemasson JJ, et al. (1997) High annual and seasonal variations in malaria transmission by anophelines and vector species composition in Dielmo, a holoendemic area in Senegal. Am J Trop Med Hyg 56: 247–253.

39. Gadiaga L, Machault V, Pagès F, Gaye A, Jarjaval F, et al. (2011) Conditions of malaria transmission in Dakar from 2007 to 2010. Malar J 10: 312.

40. Gimnig JE, Ombok M, Kamau L, Hawley W a (2001) Characteristics of larval anopheline (Diptera: Culicidae) habitats in Western Kenya. J Med Entomol 38: 282–288.

41. Gimonneau G, Pombi M, Choisy M, Morand S, Dabiré RK, et al. (2012) Larval habitat segregation between the molecular forms of the mosquito Anopheles gambiae in a rice field area of Burkina Faso, West Africa. Med Vet Entomol 26: 9–17.

42. Highton RB, Bryan JH, Boreham PFL, Chandler JA (1979) Studies on the sibling species Anopheles gambiae Giles and Anopheles arabiensis Patton (Diptera: Culicidae) in the Kisumu area, Kenya. Bull Entomol Res 69: 43–53.

43. Impoinvil D, Keating J (2007) The association between distance to water pipes and water bodies positive for anopheline mosquitoes (Diptera: Culicidae) in the urban community of Malindi,. J Vector Ecol 32: 319–327.

44. Jarju LBS, Fillinger U, Green C, Louca V, Majambere S, et al. (2009) Agriculture and the promotion of insect pests: rice cultivation in river floodplains and malaria vectors in The Gambia. Malar J 8: 170.

45. Jones CM, Sanou A, Guelbeogo WM, Sagnon N, Johnson PCD, et al. (2012) Aging partially restores the efficacy of malaria vector control in insecticide-resistant populations of Anopheles gambiae s.l. from Burkina Faso. Malar J 11: 24.

46. Kawada H, Dida GO, Ohashi K, Komagata O, Kasai S, et al. (2011) Multimodal pyrethroid resistance in malaria vectors, Anopheles gambiae s.s., Anopheles arabiensis, and Anopheles funestus s.s. in western Kenya. PLoS One 6: e22574.

47. Kweka EJ, Zhou G, Munga S, Lee M-C, Atieli HE, et al. (2012) Anopheline larval habitats seasonality and species distribution: a prerequisite for effective targeted larval habitats control programmes. PLoS One 7: e52084.

48. Le Goff G, Leong Pock Tsy JM, Robert V (2006) Molecular characterization of the malaria vector Anopheles gambiae s.s. in Madagascar. Med Vet Entomol 20: 259–260.

49. Lehmann T, Licht M, Elissa N, Maega BTA, Chimumbwa JM, et al. (2003) Population Structure of Anopheles gambiae in Africa. J Hered 94: 133–147.

50. Lemasson JJ, Fontenille D, Lochouarn L, Dia I, Simard F, et al. (1997) Comparison of behavior and vector efficiency of Anopheles gambiae and An. arabiensis (Diptera:Culicidae) in Barkedji, a Sahelian area of Senegal. J Med Entomol 34: 396–403.

51. Machault V, Vignolles C, Pagès F, Gadiaga L, Tourre YM, et al. (2012) Risk mapping of Anopheles gambiae s.l. densities using remotely-sensed environmental and meteorological data in an urban area: Dakar, Senegal. PLoS One 7: e50674.

52. Minakawa N, Mutero CM, Githure JI, Beier JC, Yan G (1999) Spatial distribution and habitat characterization of anopheline mosquito larvae in Western Kenya. Am J Trop Med Hyg 61: 1010–1016.

53. Minakawa N, Munga S, Atieli F, Mushinzimana E, Zhou G, et al. (2005) Spatial distribution of anopheline larval habitats in Western Kenyan highlands: effects of land cover types and topography. Am J Trop Med Hyg 73: 157–165.

54. Minakawa N, Seda P, Yan G (2002) Influence of host and larval habitat distribution on the abundance of African malaria vectors in western Kenya. Am J Trop Med Hyg 67: 32–38.

55. Minakawa N, Sonye G, Yan G (2005) Relationships between occurrence of Anopheles gambiae s.l. (Diptera: Culicidae) and size and stability of larval habitats. J Med Entomol 42: 295–300.

56. Mnzava a E, Kilama WL (1986) Observations on the distribution of the Anopheles gambiae complex in Tanzania. Acta Trop 43: 277–282.

57. Mosha FW, Petrarca V (1983) Ecological studies on Anopheles gambiae complex sibling species on the Kenya coast. Trans R Soc Trop Med Hyg 77: 344–345.

58. Mosha FW, Subra R (1982) Ecological studies on Anopheles gambiae complex sibling species in Kenya I. Preliminary observations on their geographical distribution and chromosomal polymorphic inversions. Bull World Health Organ VBC/82.867: 1–9.

59. Mpofu SM (1985) Seasonal vector density and disease incidence patterns of malaria in an area of Zimbabwe. Trans R Soc Trop Med Hyg 79: 169–175.

60. Munga S, Minakawa N, Zhou G, Mushinzimana E, Barrack O-OJ, et al. (2006) Association Between Land Cover and Habitat Productivity of Malaria Vectors in Western Kenyan Highlands. Am J Trop Med Hyg 74: 69–75.

61. Mwangangi JM, Mbogo CM, Muturi EJ, Nzovu JG, Githure JI, et al. (2007) Spatial distribution and habitat characterisation of Anopheles larvae along the Kenyan coast. J Vector Borne Dis 44: 44–51.

62. Ndiath MO, Brengues C, Konate L, Sokhna C, Boudin C, et al. (2008) Dynamics of transmission of Plasmodium falciparum by Anopheles arabiensis and the molecular forms M and S of Anopheles gambiae in Dielmo, Senegal. Malar J 7: 136.

63. Odiere M, Bayoh MN, Gimnig J, Vulule J, Irungu L, et al. (2007) Sampling outdoor, resting Anopheles gambiae and other mosquitoes (Diptera: Culicidae) in western Kenya with clay pots. J Med Entomol 44: 14–22.

64. Oliveira E, Salgueiro P, Palsson K, Vicente JL, Arez a P, et al. (2008) High levels of hybridization between molecular forms of Anopheles gambiae from Guinea Bissau. J Med Entomol 45: 1057–1063.

65. Onyabe DY, Vajime CG, Nock IH, Ndams IS, Akpa a U, et al. (2003) The distribution of M and S molecular forms of Anopheles gambiae in Nigeria. Trans R Soc Trop Med Hyg 97: 605–608.

66. Oyewole IO, Awolola TS (2006) Impact of urbanisation on bionomics and distribution of malaria vectors in Lagos, southwestern Nigeria. J Vector Borne Dis 43: 173–178.

67. Petrarca V, Beier JC, Onyango F, Koros J, Asiago C, et al. (1991) Species composition of the Anopheles gambiae complex (Diptera: Culicidae) at two sites in western Kenya. J Med Entomol 28: 307–313.

68. Petrarca V, Carrara GC, Di Deco MA, Petrangeli G (1983) Il complesso Anopheles gambiae in Guinea Bissau. Parassitologia: 29–39.

69. Petrarca V, Carrara GC, Di Deco MA, Petrangeli G (1984) Osservazioni citogenetiche e biometriche sui membri del complesso Anopheles gambiae in Mozambico. Parassitologia 26: 247–259.

70. Petrarca V, Nugud AD, Elkarim Ahmed MA, Haridi AM, Di Deco MA, et al. (2000) Cytogenetics of the Anopheles gambiae complex in Sudan, with special reference to An. arabiensis: relationships with East and West African populations. Med Vet Entomol 14: 149–164.

71. Petrarca V, Petrangeli G, Rossi P, Sabatinelli G (1986) Etude chromosomique d’Anopheles gambiae et Anopheles arabiensis a Ouagadougou (Burkina Faso) et dans quelques villages voisins. Parassitologia 28: 41–61.

72. Petrarca V, Sabatinelli G, Touré YT, Di Deco MA (1998) Morphometric multivariate analysis of field samples of adult Anopheles arabiensis and An. gambiae s.s. (Diptera: Culicidae). J Med Entomol 35: 16–25.

73. Petrarca V, Vercruysse J, Coluzzi M (1987) Observations on the Anopheles gambiae complex in the Senegal River Basin, West Africa. Med Vet Entomol 1: 303–312.

74. Petrarca V, Beier J (1992) Intraspecific chromosomal polymorphism in the Anopheles gambiae complex as a factor affecting malaria transmission in the Kisumu area of Kenya. Am J Trop Med Hyg 46: 229–237.

75. Reimer LJ, Tripet F, Slotman M, Spielman A, Fondjo E, et al. (2005) An unusual distribution of the kdr gene among populations of Anopheles gambiae on the island of Bioko, Equatorial Guinea. Insect Mol Biol 14: 683–688.

76. Rishikesh N, Di Deco MA, Petrarca V, Coluzzi M (1985) Seasonal variations in indoor resting Anopheles gambiae and Anopheles arabiensis in Kaduna, Nigeria. Acta Trop 42: 165–170.

77. Robert V, Petrarca V, Carnevale P, Ovazza L, Coluzzi M (1989) Analyse cytogénétique du complexe Anopheles gambiae dans la région de Bobo-Dioulasso (Burkina Faso). Ann Parasitol Hum Comparé 64: 290–311.

78. Service MW (1970) Identification of the Anopheles gambiae complex in Nigeria by larval and adult chromosomes. Ann Trop Med Parasitol 64: 131–136.

79. Service MW (1972) Identification of the Anopheles gambiae complex in the Western Nyanza area, Kenya, 1971. Bull World Health Organ VBC/72.362: 1–12.

80. Shidrawi GR (1972) The distribution and seasonal prevalence of members of the Anopheles gambiae species complex (species A & B) in Garki District, Northern Nigeria. Bull World Health Organ VBC/72.387: 1–16.

81. Simard F, Ayala D, Kamdem GC, Pombi M, Etouna J, et al. (2009) Ecological niche partitioning between Anopheles gambiae molecular forms in Cameroon: the ecological side of speciation. BMC Ecol 9: 17.

82. Stump AD, Atieli FK, Vulule JM, Besansky NJ (2004) Dynamics of the pyrethroid knockdown resistance allele in western Kenyan populations of Anopheles gambiae in response to insecticide-treated bed net trials. Am J Trop Med Hyg 70: 591–596.

83. Tantely ML, Rakotoniaina J-C, Tata E, Andrianaivolambo L, Fontenille D, et al. (2012) Modification of Anopheles gambiae distribution at high altitudes in Madagascar. J Vector Ecol 37: 402–406.

84. Tchouassi DP, Quakyi I a, Addison E a, Bosompem KM, Wilson MD, et al. (2012) Characterization of malaria transmission by vector populations for improved interventions during the dry season in the Kpone-on-Sea area of coastal Ghana. Parasit Vectors 5: 212.

85. Temu EA, Hunt RH, Coetzee M, Minjas JN, Shiff CJ (1997) Detection of hybrids in natural populations of the Anopheles gambiae complex by the rDNA-based, PCR method. Ann Trop Med Parasitol 91: 963–965.

86. Touré YT, Petrarca V, Traoré SF, Coulibaly a, Maiga HM, et al. (1998) The distribution and inversion polymorphism of chromosomally recognized taxa of the Anopheles gambiae complex in Mali, West Africa. Parassitologia 40: 477–511.

87. Tripet F, Touré YT, Taylor CE, Norris DE, Dolo G, et al. (2001) DNA analysis of transferred sperm reveals significant levels of gene flow between molecular forms of Anopheles gambiae. Mol Ecol 10: 1725–1732.

88. Tripet F, Dolo G, Lanzaro G (2005) Multilevel analyses of genetic differentiation in Anopheles gambiae ss reveal patterns of gene flow important for malaria-fighting mosquito projects. Genetics 169: 313–324.

89. Vezenegho SB, Brooke BD, Hunt RH, Coetzee M, Koekemoer LL (2009) Malaria vector composition and insecticide susceptibility status in Guinea Conakry, West Africa. Med Vet Entomol 23: 326–334.

90. Wang-Sattler R, Blandin S, Ning Y, Blass C, Dolo G, et al. (2007) Mosaic genome architecture of the Anopheles gambiae species complex. PLoS One 2: e1249.

91. White G, Rosen P (1973) Comparative studies on sibling species of the Anopheles gambiae Giles complex (Dipt., Culicidae). II. Ecology of species A and B in savanna around Kaduna, Nigeria,. Bull Entomol Res 62: 613–615.

92. Wondji C, Simard F, Fontenille D (2002) Evidence for genetic differentiation between the molecular forms M and S within the Forest chromosomal form of Anopheles gambiae in an area of sympatry. Insect Mol Biol 11: 11–19.

93. Wondji C, Frédéric S, Petrarca V, Etang J, Santolamazza F, et al. (2005) Species and populations of the Anopheles gambiae complex in Cameroon with special emphasis on chromosomal and molecular forms of Anopheles gambiae s.s. J Med Entomol 42: 998–1005.

94. Yawson a E, McCall PJ, Wilson MD, Donnelly MJ (2004) Species abundance and insecticide resistance of Anopheles gambiae in selected areas of Ghana and Burkina Faso. Med Vet Entomol 18: 372–377.
