# Supplementary material for: Dissecting functional components of reproductive isolation among closely related sympatric species of the Anopheles gambiae complex
Source: Evol Appl. 2017 Oct 5;10(10):1102–20. doi: 10.1111/eva.12517 (PMC5680640; doi:10.1111/eva.12517)
Supplement: Supplementary file 3 [file EVA-10-1102-s003.docx]

## Supplementary Material

**Tables S1-S4.** Frequency of hybrids recorded in samples of natural mosquito populations from published material. African countries are classified as belonging to the Western or Eastern region according to their location relative to the Great Rift Valley system, which represents a major geographical barrier structuring *An. gambiae* *sensu lato* populations. The number of surveys refers essentially to the number of localities where collections were carried out; in some instances, however, the same locality was surveyed more than once in different years or seasons. The total number of studies is not the sum of the number of studies referred to individual countries because a few studies covered more than one country.

**Table S1. Frequency of *Anopheles arabiensis* × *gambiae* hybrids in samples of adult female mosquitoes from continental Africa.**

| **Region** | **Country** | **No. Hybrids (%)** | | **No. Specimens** | **No. Surveys** | **No. Studies** |
| --- | --- | --- | --- | --- | --- | --- |
| West Africa | Burkina Faso | 3 | (0.026%) | 11,337 | 498 | 9 |
|  | Cameroon | 0 | (0.000%) | 3,287 | 155 | 3 |
|  | Gambia | 1 | (0.009%) | 10,595 | 53 | 3 |
|  | Ghana | 0 | (0.000%) | 924 | 4 | 2 |
|  | Guinea Bissau | 0 | (0.000%) | 87 | 1 | 1 |
|  | Guinea Conakry | 0 | (0.000%) | 156 | 1 | 1 |
|  | Mali | 3 | (0.016%) | 18,415 | 97 | 6 |
|  | Nigeria | 6 | (0.040%) | 15,000 | 82 | 7 |
|  | Senegal | 0 | (0.000%) | 56,525 | 36 | 11 |
|  | *Total West Africa* | 13 | (0.011%) | 116,354 | 689 | 43 |
| East Africa | Burundi | 1 | (0.152%) | 658 | 1 | 1 |
|  | Kenya | 1 | (0.009%) | 11,027 | 45 | 11 |
|  | Madagascar | 0 | (0.000%) | 530 | 14 | 4 |
|  | Mozambique | 0 | (0.000%) | 184 | 6 | 1 |
|  | Sudan | 0 | (0.000%) | 277 | 3 | 1 |
|  | Tanzania | 2 | (0.035%) | 5,664 | 10 | 2 |
|  | Zambia | 0 | (0.000%) | 203 | 17 | 1 |
|  | Zimbabwe | 0 | (0.000%) | 107 | 1 | 1 |
|  | *Total East Africa* | 4 | (0.022%) | 18,650 | 97 | 22 |
| Grand Total |  | 17 | (0.015%) | 135,004 | 786 | 65 |

**Table S2. Frequency of *Anopheles coluzzii* × *gambiae s.s.* hybrids in samples of adult female mosquitoes from West and Central Africa.**

| **Region** | **Country** | **No. Hybrids (%)** | | **No. Specimens** | **No. Surveys** | **No. Studies** |
| --- | --- | --- | --- | --- | --- | --- |
| Central Africa | Angola | 0 | (0.00%) | 499 | 5 | 3 |
|  | Cameroon | 0 | (0.00%) | 2,577 | 61 | 7 |
|  | Central African Republic | 0 | (0.00%) | 91 | 1 | 1 |
|  | Congo | 1 | (1.92%) | 52 | 1 | 1 |
|  | Democratic Republic of Congo | 0 | (0.00%) | 47 | 1 | 1 |
|  | Equatorial Guinea | 0 | (0.00%) | 108 | 2 | 2 |
|  | *Total Central Africa* | 1 | (0.03%) | 3,374 | 71 | 15 |
| West Africa | Benin | 1 | (0.50%) | 202 | 5 | 2 |
|  | Burkina Faso | 44 | (0.66%) | 6,653 | 267 | 13 |
|  | Ghana | 4 | (0.21%) | 1,868 | 19 | 7 |
|  | Guinea Conakry | 2 | (0.39%) | 516 | 4 | 2 |
|  | Ivory Coast | 1 | (0.44%) | 226 | 7 | 2 |
|  | Mali | 1 | (0.08%) | 1,205 | 14 | 6 |
|  | Niger | 0 | (0.00%) | 18 | 1 | 1 |
|  | Nigeria | 0 | (0.00%) | 2,432 | 11 | 2 |
|  | *Total West Africa* | 53 | (0.40%) | 13,120 | 328 | 35 |
| High Hybridization | Gambia | 44 | (2.46%) | 1,789 | 24 | 3 |
| Area (HHA) | Guinea Bissau | 37 | (21.51%) | 172 | 2 | 1 |
|  | Senegal | 25 | (2.11%) | 1,185 | 10 | 3 |
|  | *Total HHA* | 106 | (3.37%) | 3,146 | 36 | 7 |
|  | Grand Total | 160 | (0.81%) | 19,640 | 435 | 57 |

**Table S3. Frequency of *Anopheles arabiensis* × *gambiae* hybrids in larval samples from continental Africa.**

| **Region** | **Country** | **No. Hybrids (%)** | | **No. Specimens** | **No. Surveys** | **No. Studies** |
| --- | --- | --- | --- | --- | --- | --- |
| West Africa | Angola | 0 | (0.00%) | 22 | 1 | 1 |
|  | Gambia | 0 | (0.00%) | 563 | 6 | 3 |
|  | Mali | 0 | (0.00%) | 1,034 | 1 | 1 |
|  | Nigeria | 0 | (0.00%) | 3,075 | 13 | 4 |
|  | *Total West Africa* | 0 | (0.00%) | 8,211 | 22 | 10 |
| East Africa | Kenya | 0 | (0.00%) | 9,315 | 31 | 11 |
|  | *Total East Africa* | 0 | (0.00%) | 9,315 | 31 | 11 |
| Grand Total |  | 0 | (0.00%) | 17,526 | 53 | 21 |

**Table S4. Frequency of *Anopheles coluzzii* × *gambiae s.s.* hybrids in larval samples from West Africa.**

| **Country** | **No. Hybrids (%)** | | **No. Specimens** | **No. Surveys** | **No. Studies** |
| --- | --- | --- | --- | --- | --- |
| Ghana | 0 | (0.00%) | 237 | 2 | 2 |
| Mali | 4 | (0.57%) | 701 | 1 | 1 |
| Nigeria | 0 | (0.00%) | 264 | 6 | 1 |
| Total | 4 | (0.33%) | 1,202 | 9 | 4 |
