# Supplementary material for: Dissecting functional components of reproductive isolation among closely related sympatric species of the Anopheles gambiae complex
Source: Evol Appl. 2017 Oct 5;10(10):1102–20. doi: 10.1111/eva.12517 (PMC5680640; doi:10.1111/eva.12517)
Supplement: Supplementary file 4 [file EVA-10-1102-s004.docx]

**Table S5. Frequency of homospecific and heterospecific insemination in *Anopheles* *gambiae s.l.* taxa from Burkina Faso.**

|  | | **Female** | | | | | **Homospecific Mating (%)** |
| --- | --- | --- | --- | --- | --- | --- | --- |
|  |  | *arabiensis* | *coluzzii* | *gambiae s.s.* | *arabiensis* x *coluzzii* | Total |  |
| **Sperm** | *arabiensis* | 317 | 1 | 5 | 1 | 324 | 97.8 |
|  | *coluzzii* | 3 | 409 | 16 | 1 | 429 | 95.3 |
|  | *gambiae s.s.* | 2 | 18 | 217 | 1 | 238 | 91.2 |
|  | absent | 38 | 14 | 23 | 0 | 75 |  |
|  | Total | 360 | 442 | 261 | 3 | 1 066 |  |
| **Insemination (%)** | | 89.4 | 96.8 | 91.2 | 100.0 | 93.0 |  |
