# Supplementary material for: Dissecting functional components of reproductive isolation among closely related sympatric species of the Anopheles gambiae complex
Source: Evol Appl. 2017 Oct 5;10(10):1102–20. doi: 10.1111/eva.12517 (PMC5680640; doi:10.1111/eva.12517)
Supplement: Supplementary file 5 [file EVA-10-1102-s005.docx]

**Table S6. Analysis of Deviance of generalized linear models testing the effect of frequency-dependent hybridization in *An. arabiensis*, *An. coluzzii*, and *An. gambiae* s.s., based on the strength of their pair sexual isolation indices *I*_PSI_.** The parameter estimates of the minimal adequate model (No. 1, containing all main effects and their interaction) are given in Table 2.

| **Model Terms** | **Residual Deviance** | **AIC** | **Change in Deviance** | **d.f.** | **F-test** | ***P*** |  |
| --- | --- | --- | --- | --- | --- | --- | --- |
|  | | | | | | | |
| 1. Frequency ☓ Taxa | 0.001131 | –48.492 |  |  |  |  |  |
| 2. Frequency + Taxa | 0.008556 | –32.258 | 0.007425 | 2 | 13.128 | 0.017 |  |
| 3. Taxa | 0.016756 | –27.537 | 0.008200 | 1 | 5.750 | 0.053 |  |
| 4. Frequency | 0.028610 | –24.187 | 0.020054 | 2 | 7.031 | 0.027 |  |
| 5. Null Model | 0.029052 | –26.034 | 0.020496 | 3 | 4.791 | 0.049 |  |
