# Supplementary material for: Dissecting functional components of reproductive isolation among closely related sympatric species of the Anopheles gambiae complex
Source: Evol Appl. 2017 Oct 5;10(10):1102–20. doi: 10.1111/eva.12517 (PMC5680640; doi:10.1111/eva.12517)
Supplement: Supplementary file 6 [file EVA-10-1102-s006.docx]

**Table S7. Frequency of *Anopheles coluzzii* × *gambiae s.s.* hybrids in larval samples of populations from the rainforest eco-climatic domain of Cameroon, Central Africa**.

| **Locality (altitude) / Period** | **No. Hybrids (%)** | | **No. *An. coluzzii*** | **No. *An. gambiae*** | | **No. Surveys** | |
| --- | --- | --- | --- | --- | --- | --- | --- |
| *Guinean side of CVL* | | | | | | |  |
| Ekondo Titi Beach (10 m) – Dec 2012 | 0 | (0.00%) | 79 | 4 | 1 | |  |
| Ekondo Titi Town (45 m) – Dec 2012 | 0 | (0.00%) | 6 | 112 | 1 | |  |
| Idenau (8 m) – Oct 2012 | 0 | (0.00%) | 26 | 0 | 1 | |  |
| Kumba (210-263 m) – Mar 2010 | 0 | (0.00%) | 4 | 182 | 1 | |  |
| Mamfe (49-94 m) – Mar 2010 | 0 | (0.00%) | 14 | 159 | 1 | |  |
| Nguti (230 m) – Mar 2010 | 0 | (0.00%) | 1 | 86 | 1 | |  |
| *Total Guinean* | 0 | (0.00%) | 130 | 543 | 6 | |  |
| *Cameroon Volcanic Line (CVL)* | | | | | | |  |
| Buea (480-790 m) – Mar 2010 | 2 | (0.97%) | 75 | 129 | 1 | |  |
| Loum (218-263 m) – Apr 2010 | 0 | (0.00%) | 45 | 9 | 1 | |  |
| Mbanga (116-123 m) – Apr 2010 | 0 | (0.00%) | 67 | 77 | 1 | |  |
| Njombe (88-94 m) – Apr 2010 | 0 | (0.00%) | 36 | 1 | 1 | |  |
| Nkongsamba (800-924 m) – Apr 2010 | 0 | (0.00%) | 134 | 8 | 1 | |  |
| Tibati (850-880 m) – Aug 2012 | 1 | (0.22%) | 12 | 433 | 1 | |  |
| Tibati (850-880 m) – Sep 2012 | 2 | (0.20%) | 23 | 962 | 1 | |  |
| Tibati (850-880 m) – Oct 2012 | 1 | (0.44%) | 3 | 221 | 1 | |  |
| *Total CVL* | 6 | (0.27%) | 395 | 1,840 | 8 | |  |
| *Congolese side of CVL* | | | | | | |  |
| Bouandjo (4 m) – Jun 2010 | 0 | (0.00%) | 34 | 19 | 1 | |  |
| Bwambe (8-24 m) – Jun 2010 | 0 | (0.00%) | 60 | 61 | 1 | |  |
| Campo (0-20 m) – Jun 2010 | 0 | (0.00%) | 157 | 121 | 1 | |  |
| Douala (0-50 m) – Mar 2010 | 0 | (0.00%) | 123 | 8 | 1 | |  |
| Ebodje (0-4 m) – Jun 2010 | 0 | (0.00%) | 239 | 12 | 1 | |  |
| Eboundja (27 m) – Jun 2010 | 0 | (0.00%) | 1 | 75 | 1 | |  |
| Grand Batanga (10-23) – Jun 2010 | 0 | (0.00%) | 73 | 15 | 1 | |  |
| Kribi (4-20 m) – Jun 2010 | 0 | (0.00%) | 359 | 52 | 1 | |  |
| Lolabe (0 m) – Jun 2010 | 0 | (0.00%) | 30 | 66 | 1 | |  |
| Nziou (4-20 m) – Jun 2010 | 0 | (0.00%) | 251 | 25 | 1 | |  |
| Tiko (8-49 m) – Mar 2010 | 0 | (0.00%) | 116 | 4 | 1 | |  |
| Yaoundé (700-760 m) – May 2008 | 0 | (0.00%) | 173 | 130 | 14 | |  |
| Yaoundé (700-760 m) – Jun 2008 | 0 | (0.00%) | 139 | 72 | 12 | |  |
| Yaoundé (700-760 m) – Jul 2008 | 1 | (0.36%) | 175 | 98 | 13 | |  |
| Yaoundé (700-760 m) – Aug 2008 | 1 | (0.55%) | 108 | 73 | 12 | |  |
| Yaoundé (700-760 m) – Sep 2008 | 1 | (0.32%) | 243 | 72 | 15 | |  |
| Yaoundé (700-760 m) – Oct 2008 | 1 | (0.20%) | 369 | 134 | 15 | |  |
| Yaoundé (700-760 m) – Nov 2008 | 0 | (0.00%) | 201 | 20 | 11 | |  |
| Yaoundé (700-760 m) – Dec 2008 | 0 | (0.00%) | 185 | 30 | 14 | |  |
| Yaoundé (700-760 m) – Jan 2009 | 0 | (0.00%) | 196 | 43 | 10 | |  |
| Yaoundé (700-760 m) – Feb 2009 | 0 | (0.00%) | 277 | 120 | 12 | |  |
| Yaoundé (700-760 m) – Mar 2009 | 0 | (0.00%) | 114 | 46 | 9 | |  |
| Yaoundé (700-760 m) – Apr 2009 | 0 | (0.00%) | 194 | 70 | 12 | |  |
| Yoyo – Dec 2012 | 0 | (0.00%) | 78 | 0 | 1 | |  |
| *Total Congolese* | 4 | (0.08%) | 3,895 | 1,366 | 160 | |  |
| **Grand Total** | 10 | (0.12%) | 4,420 | 3,749 | 174 | |  |
